# Novel findings to the biosynthetic pathway of magnoflorine and taspine through transcriptomic and metabolomic analysis of *Croton draco* (Euphorbiaceae)

**DOI:** 10.1186/s12870-019-2195-y

**Published:** 2019-12-18

**Authors:** Anahí Canedo-Téxon, Feliza Ramón-Farias, Juan Luis Monribot-Villanueva, Emanuel Villafán, Alexandro Alonso-Sánchez, Claudia Anahí Pérez-Torres, Guillermo Ángeles, José Antonio Guerrero-Analco, Enrique Ibarra-Laclette

**Affiliations:** 1 Instituto de Ecología A.C., Red de Estudios Moleculares Avanzados, 91070 Xalapa, Veracruz, México; 2 Universidad Veracruzana (Campus Peñuela-Córdoba), Amatlán de los Reyes, 94945 Veracruz, México; 3 Catedrático CONACyT en el Instituto de Ecología A.C, Veracruz, México; 4 Instituto de Ecología A.C., Red de Ecología Funcional, 91070 Xalapa, Veracruz, México

**Keywords:** *Croton draco*, Aporphine alkaloids, Magnoflorine, Taspine, RNA-seq

## Abstract

**Background:**

*Croton draco* is an arboreal species and its latex as well as some other parts of the plant, are traditionally used in the treatment of a wide range of ailments and diseases. Alkaloids, such as magnoflorine, prevent early atherosclerosis progression while taspine, an abundant constituent of latex, has been described as a wound-healer and antitumor-agent. Despite the great interest for these and other secondary metabolites, no omics resources existed for the species and the biosynthetic pathways of these alkaloids remain largely unknown.

**Results:**

To gain insights into the pathways involved in magnoflorine and taspine biosynthesis by *C. draco* and identify the key enzymes in these processes, we performed an integrated analysis of the transcriptome and metabolome in the major organs (roots, stem, leaves, inflorescences, and flowers) of this species. Transcript profiles were generated through high-throughput RNA-sequencing analysis while targeted and high resolution untargeted metabolomic profiling was also performed. The biosynthesis of these compounds appears to occur in the plant organs examined, but intermediaries may be translocated from the cells in which they are produced to other cells in which they accumulate.

**Conclusions:**

Our results provide a framework to better understand magnoflorine and taspine biosynthesis in *C. draco*. In addition, we demonstrate the potential of multi-omics approaches to identify candidate genes involved in the biosynthetic pathways of interest.

## Background

*Croton draco* var. *draco* is an arboreal species that belongs to the Euphorbiaceae family, which is widely distributed across the Americas. It is commonly known as ‘sangregado’, ‘palo sangriento’ (bleeding tree), ‘llora sangre’ (blood weeper) or, ‘sangre de dragon’ (dragon’s blood) due to the intense-red latex that these trees produce [1]. As for other latex-bearing plants, laticifer cells have been described for *Croton* species [1]. These living specialized cells synthetize and store latex in the form of a tubular system, independent of the xylem and phloem [2]. The aspects of laticifer cells that have attracted most attention, are those related to their physiology and their role in the production of latex, particularly in the rubber tree (*Hevea brasiliensis*), opium poppy (*Papaver somniferum*), and in some species of the *Croton* genus [1, 3–6]. Laticifers are involved in plant defence against herbivores and the transport of some secondary metabolites [7–9], although their precise ontogenic origin, their mechanisms of differentiation and the organization of the laticifer system which often is present in several organs of the plants, remain uncertain.

*C. draco* is traditionally used in the treatment of a wide range of ailments and diseases. The medicinal properties of the plant can be attributed to the abundance of secondary metabolites present in all of its organs which include alkaloids, tannins, diterpenes, and essential oils [10–12]. Latex has been used frequently in traditional medicine and its chemical constituents have been characterised in several species of *Croton*. The most abundant secondary metabolites (up to 90% of the dry weight) are proanthocyanidins and several polyphenolic compounds such as (+)-catechin, (−)-epicatechin, gallocatechin, epigallocatechin and dimeric procyanidins [13]. Other minor constituents have also been identified including korberins [14] such as bincatriol, crolechinol, and crolechinic acid [15] among others [16, 17]. In *C. lechleri*, one of the most studied species of the genus, the alkaloid taspine was also identified as an abundant constituent of latex, representing approximately 9% of its dry weight [18]. Taspine is a benzylisoquinoline-type alkaloid that has been described as a wound-healer [19] and antitumor agent [20]. The latter property has been demonstrated experimentally using latex and the taspine isolated from *C. lechleri* [21], and similar effects have been reported for other taspine derivatives tested against several cancer cell lineages [22, 23]. Although the biosynthetic pathway of taspine remains unknown, its synthesis in some plant species involves enzymatic reactions that catalyse the Hofmann elimination of (+)-magnoflorine (a quaternary aporphine) leading to the formation of magnoflorine methine and the oxidation of the double bond between C9 and C10 and its lactonization that finally produces taspine [24]. The presence of magnoflorine has been detected in the related species *C. cumingii* [25], *C. xalapensis* [26] and *C. lechleri* [18].

Despite the pharmacological potential of the *Croton* genus, there are no genomic data available for these species and the complete pathways for the biosynthesis of taspine and other secondary metabolites remain unknown. Therefore, we performed the sequencing and de novo assembly of RNA libraries generated from distinct organs collected from well-established *C. draco* trees which had been propagated by bud grafting. Also, we provide an annotation to public databases and categorize the transcripts according to their functions and metabolic pathways. Finally, based on these results along with those corroborated by untargeted and targeted metabolomic analysis performed by Liquid Chromatography-High Resolution Mass Spectrometry (LC-HRMS) and Liquid Chromatography-tandem Mass Spectrometry (LC-MS-MS), respectively; a taspine biosynthetic pathway is suggested and, for the first time, a number of unigenes which encode enzymes involved in this pathway have been identified.

## Results

### Construction of the unigenes set for *Croton draco*

A total of 87,884,170 raw Illumina paired-end reads were generated from all organ samples (leaves, stems, roots, fruits, inflorescences and flowers). 7.78 to 19.4 million sequences per sample, with an average of 14.6 million per sample were generated. Around 16.79% of the raw Illumina paired-end reads were discarded because of inadequate quality (see method for more details). To improve computing time and quality, prior to assembly, longer reads were generated by joining paired reads through their overlapping regions (Additional file 1: Table S1). Both datasets (long reads and paired-end reads that were not merged) were used to assemble the transcriptome of *C. draco* that comprises a total of 441,543 unigenes, ranging from 200 to 17,193 bp with an average length of 691.32 bp (Additional file 2 and Additional file 3).

As the *C. draco* transcriptome was generated using RNA-seq methodology based on poly(A) selection, the coding sequences (ranging between 21 and 16,344 nucleotides) were identified and translated once frameshifts produced by erroneous insertions/deletions had been corrected (266,967 unigenes, 60.46% of the total) using a homology-based method performed using the AlingWise pipeline [27]. It is likely that the remaining sequences represented untranslated regions (UTRs) or non-coding RNAs (ncRNAs). However, is also possible that these represent sequences with several common assembly artefacts, including chimer, spurious insertions in contigs, and structural abnormalities such as incompleteness, fragmentation, or local misassembly of contigs. Since the likelihood of finding similarity to previously described proteins is dependent on the query sequence length [28], only non-redundant coding sequences of at least 75 amino acids, were annotated and used in future analyses (a total of 174,925 unigenes; Additional files 2 and 3; Additional file 4: Figure S1 and Additional file 5: Table S2).

### Homology search and functional annotation of *C. draco* unigenes

**Fig. 1:**
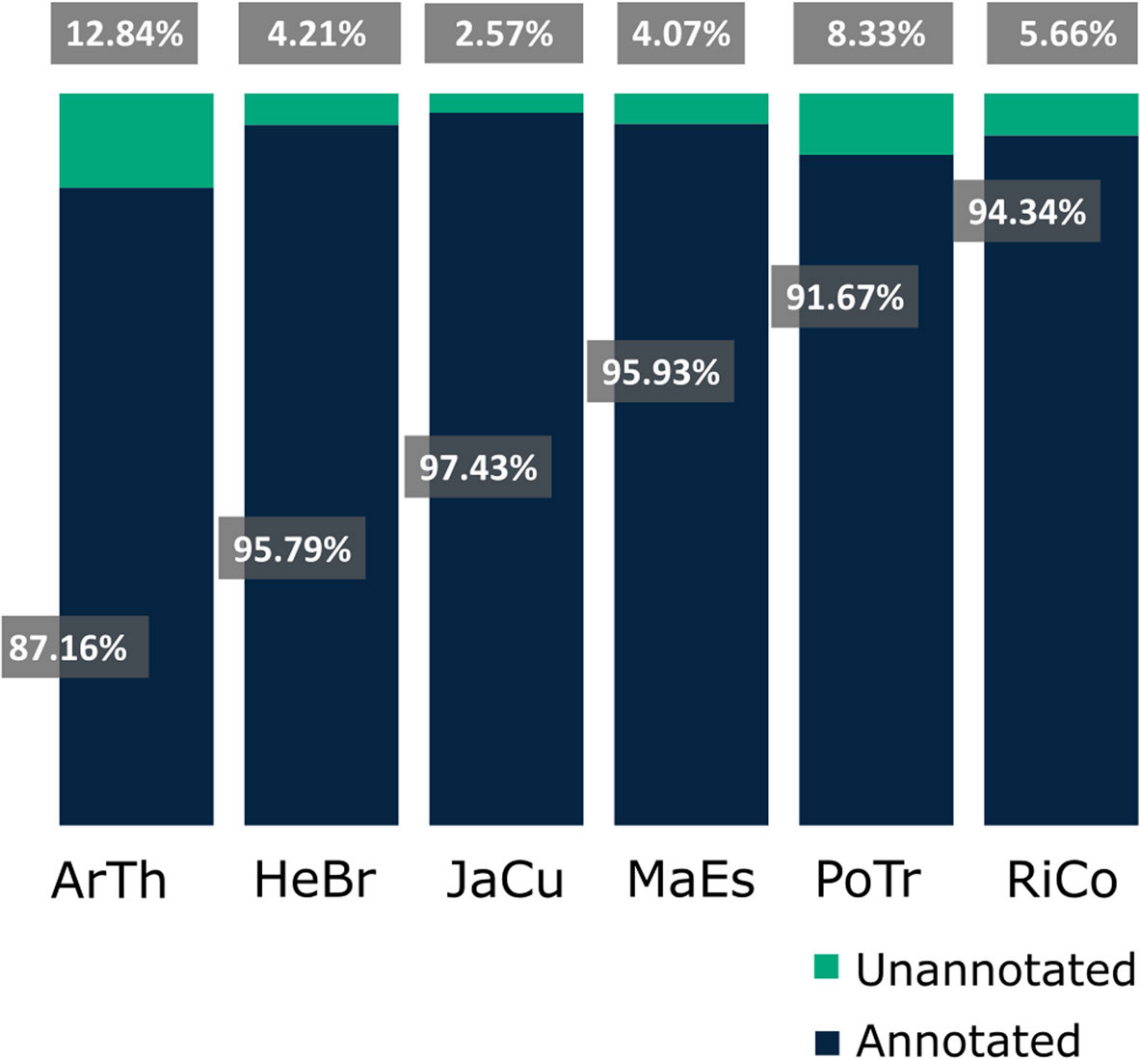
Percentage of annotated *C. draco* genes by homologous protein identification from other plant species. BLASTp similarity searches were performed using the translated CDS identified in *C. draco* non-redundant unigenes. Reference proteomes correspond to angiosperm plant species that belong to the Malpighiales order [*Hevea brasiliensis* (HeBr), *Jatropha curcas* (JaCu), *Manihot esculenta* (MaEs), *Populus trichocarpa* (PoTr), and *Ricinus communis* (RiCo). *Arabidopsis thaliana* (ArTh), order Brassicales, was also included in the annotation process

We determined the *C. draco* proteins that could be annotated by BLAST similarity using selected proteomes (five angiosperms from the Malpighiales order and *Arabidopsis thaliana*). Most of the corresponding proteins (98.20%), showed high similarity (e-value ≤10^− 5^) with proteins from at least one of the reference species (Additional file 5: Table S2). From the total of *C. draco* non-redundant unigenes (167,071), 97.43% were annotated based on *Jatropha curcas* homologous proteins. Homologous proteins were also detected with respect to *Manihot esculenta* (95.93%), *Hevea brasiliensis* (95.79%), *Ricinus communis* (94.34%), *Populus trichocarpa* (91.67%), and *Arabidopsis thaliana* (87.16%) (Fig. 1). Counting the unique accession numbers (or locus identifiers) corresponding to the reference proteins identified as homologous to the *C. draco* unigenes, we estimate the number of unique genes from the *C. draco* genome that are represented in the transcriptome. These numbers varied between 16,942 and 24,544 proteins depending on the species, which represented the most stringent estimation of the minimal number of genes found and expressed in the *C. draco* organs sampled (Additional file 5: Table S2). We further compared *C. draco* unigenes against the Pfam domains database [29]. A total of 116,981 *C. draco* unigenes contained at least one Pfam domain and 6391 distinct Pfam domains were represented by the unigenes (Additional file 5: Table S2).

**Fig. 2:**
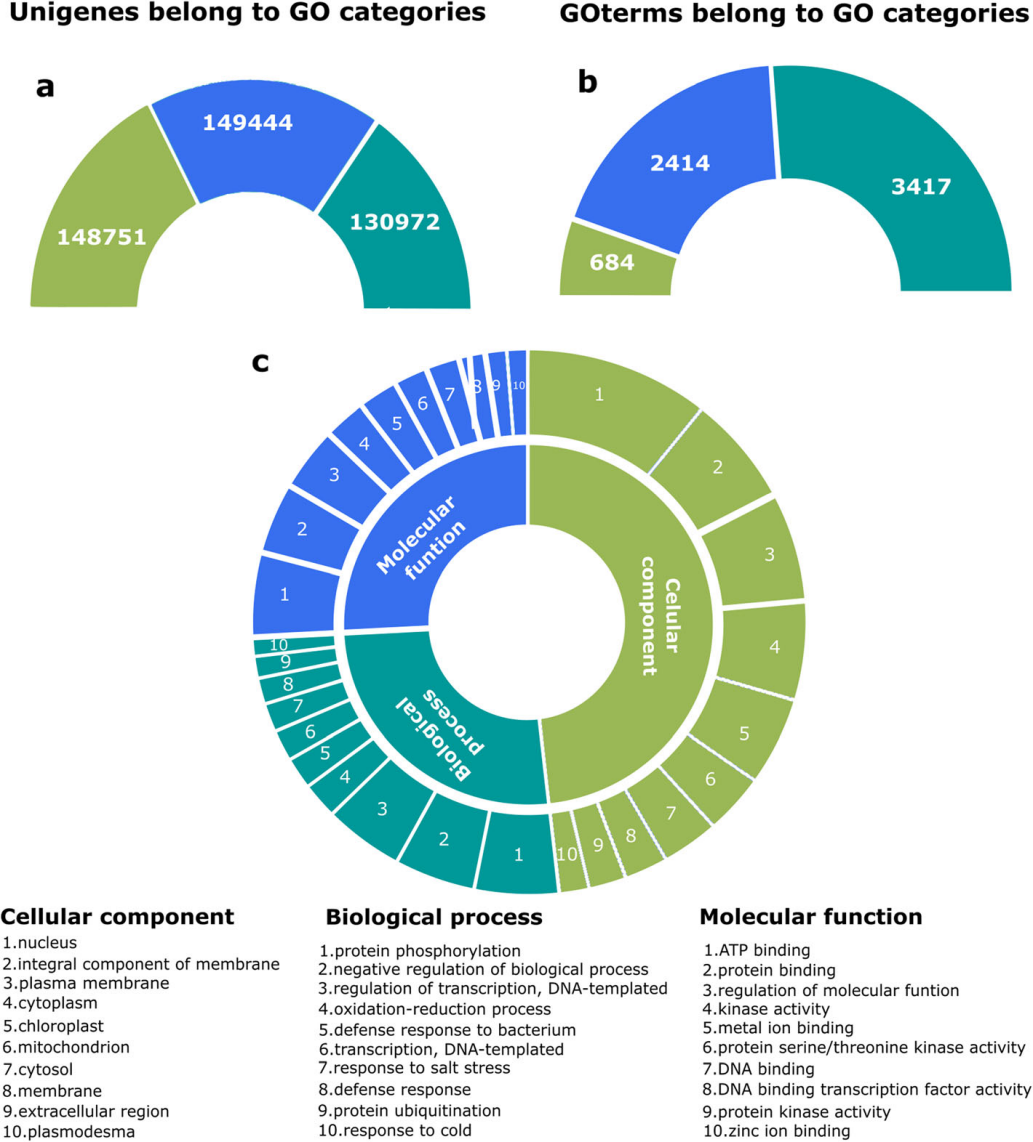
Gene ontology functional characterization of *C. draco* unigenes. Unigenes that were annotated (**a**) were classified based on the assignation of at least one of the unique GO-terms (**b**) that belongs to the three major categories (cellular component, molecular function and biological process). Slices in (**c**) panel show top ten most represented GO terms from each category. The number in brackets refers to the total number of unigenes that were assigned to each GO category

The Gene Ontology (GO) functional characterization [30] was based on the information for the corresponding homologs in the *Arabidopsis* proteome. Around 6515 unique GO terms were assigned to a total of 149,444 *C. draco* unigenes, including 3417 GO terms from the ‘biological process’ category, 2414 from ‘molecular function’ and, 684 from ‘cellular component’ (Fig. 2a and b). On average, more than eight GO-terms were allocated to each of the *C. draco* unigenes (Additional file 6: Table S3). The most prominent GO terms in the categories biological process, molecular function and cellular component were protein phosphorylation, ATP binding and nucleus, respectively (Fig. 2c).

Finally, assignments of the *C. draco* genes to some canonical metabolic pathways was performed by BLAST comparison against the KEGG database using the bidirectional best-hit method, resulting in the assignment of 1958 unique KEGG orthology (KO) identifiers and 1023 distinct enzyme commission (EC) numbers to a total of 38,514 *C. draco* unigenes (Additional file 6: Table S3). Representativeness and completeness of the assembled transcriptome were assessed by location of the KO identifiers assigned to *C. draco* unigenes into a global map of metabolic pathways. Metabolic pathways represented in the unigenes set was compared against those present in the genome of *Arabidopsis thaliana* and discrete differences were identified (Additional file 5: Figure S2). We quantified completeness of assembled transcriptome which was estimated on 81.3% based on the identification of the predefined set of 1375 embryophyte single copy orthologs. This was accomplished using the BUSCO v3.0.1 (Benchmarking Universal Single-Copy Orthologs) pipeline [31]. Together, these data suggest that the transcriptome comprises a broad coverage of the protein coding-genes present in the *C. draco* genome.

### Gene expression atlas of multiple *C. draco* organs

A transcriptional atlas was generated containing the expression profiles from each of the unigenes of the major plant organs of *C. draco*. This dataset is important because it can be used to establish the identity of each organ (Additional file 7: Table S4). Preferentially expressed genes were defined as those unigenes in which organ-specific transcript levels were at least double (logFC ≥1) that of any other organ and this different was statistically significant at FDR adjusted *P* ≤ 0.001.

**Fig. 3:**
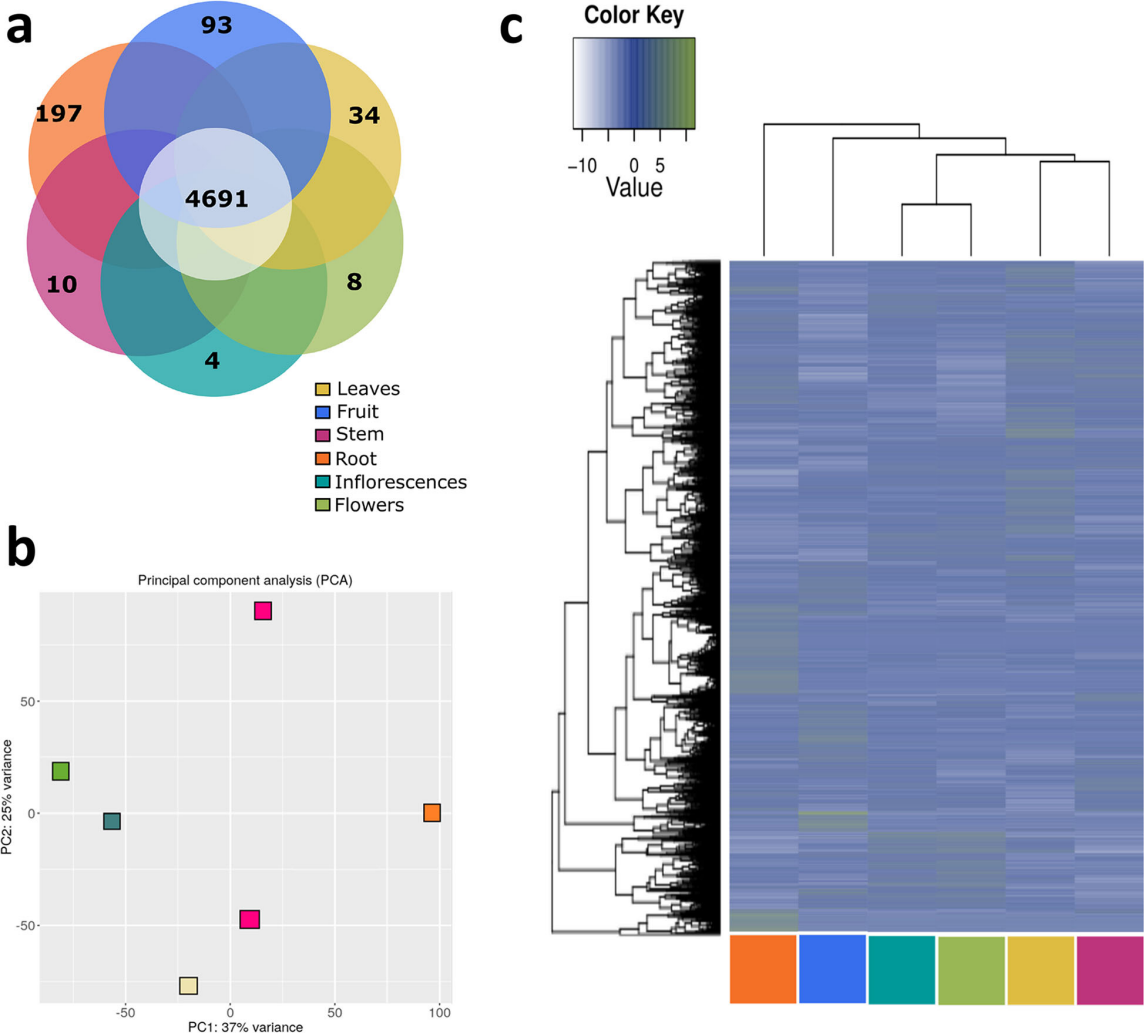
A based gene expression (RNA-seq) transcriptional atlas generated for the major organs of *C. draco*. **a** Venn diagram of the preferentially expressed unigenes in which solely those consider to be organ-specific and ubiquitous are represented. **b** Principal component analysis (PCA) of global transcriptional profiles. **c** Hierarchical clustering (HC) based on FPKM values calculated for *C. draco* unigenes preferentially expressed in some of the major organs analysed (leaves, fruits, stems, roots, inflorescences, and flowers). The dark blue represents higher expression values while the lowest values are shown in white

A total of 10,556 *C. draco* unigenes were identified as preferentially expressed in at least one of the organs (Additional file 7: Table S5). Some of these may be considered organ-specific genes because all the reads mapped to these unigenes were derived from a single library (Additional file 7: Table S5). The remaining genes, despite their preferential expression, may also be considered as ubiquitous because they were transcribed at levels over threefold above the background value (FPKM values ≥3), in all the organs analysed (Fig. 3a). Despite the majority of preferentially expressed *C. draco* unigenes (~ 93% of the total) were classified as ubiquitous, their expression profiles allow classified according to principal component analysis (PCA) and by hierarchical clustering (HC) (Fig. 3b and c, respectively).

For the organ types sampled, both the HC and the PCA reflect the known similarity of biological functions among organs. For example, transcripts from stems and leaves clustered as a neighbouring group, reflecting their physiological similarity (vegetative organs). Likewise, the reproductive organ transcriptomes (inflorescences and flowers) were grouped together, whereas gene products of roots and fruits are maintained as independents and separately from the rest.

For each analysed organ, we surveyed those categories enriched with significant numbers of preferentially expressed genes. Interestingly, these GO categories were related to specific functional roles performed by each organ (Additional file 4: Figure S3). For the primary organs such as stem, root, and leaf, enrichment categories were related mainly to plant nutrition (e.g. transport of ions, peptides and hormone transport), whereas in the inflorescences and flowers, the overrepresented categories were mainly related to the development of reproductive organs (e.g., flower, stamen, carpel, whorl and pollen).

Surveys of some specific genes produced interesting results. For example, some of the *C. draco* unigenes (UniGene120823 and UniGene211389), which are homologs to *Arabidopsis* proteins related to the light-harvesting complex II (AT1G29930 and AT3G61470, respectively), were preferentially expressed in leaves. These proteins are implicated in the regulation of excitation energy distribution between Photosystem I subunit F (AT1G31330) and subunit L (AT4G12800), (UniGene104983 and UniGene91612, respectively) [32] and Photosystem II (an extrinsic subunit of PS II, AT5G66570| UniGene92418) [33]. Similar expression profiles were found in members of the *Arabidopsis* RBCS multigene family (AT1G67090| UniGene120289), which produces RuBisCo (ribulose-1,5-bisphosphate carboxylase/oxygenase) for photosynthetic activity [34]. All the proteins mentioned above are codified by genes that are expressed specifically in *Arabidopsis* leaves [35]. The *C. draco* unigene UniGene70470, which encodes a homolog of the *Arabidopsis* desiccation-related protein (AT1G47980), is expressed in flowers. In *Arabidopsis,* the gene *At1g47980* is expressed in flower, petal, seed, sepal and stamen during the flowering, petal differentiation and expansion stages. In addition, it has been reported that *mia* (*male gametogenesis impaired anthers*) insertion mutants, which produce fragile pollen grains that fail to dissociate from tetrads, have markedly increased expression of the *At1g47980* gene [36]. Likewise, in fruits, the *C. draco* gene (UniGene37221), which encodes a homolog of CRUCIFERINA (AT5G44120), was expressed in an organ-specific manner. This was predictable since cruciferin gene families are expressed in a coordinated manner but with tissue-specific differences during seed filling and development [37–39]. Moreover, in the list of *C. draco* unigenes that are expressed solely in roots, there was a group homologous to several transcription factors that have been reported previously in *Arabidopsis*, and that are specific to roots (AT1G21860|UniGene25896, AT1G66350|UniGene17133, AT1G74150|UniGene54724, AT2G27880|UniGene6800, AT3G06460|UniGene78483, AT3G45300|UniGene68073, and AT3G47570|UniGene14449) [40].

To validate the expression profiles obtained by normalized read counts (FPKM), an RT-qPCR analysis was performed for ten of the unigenes discussed above. All the unigenes evaluated showed RT-qPCR expression profiles in complete agreement with the profiles calculated through the read counts analyses (Additional file 7: Figure S4).

### Identification of the ortholog groups across compared plant species

**Fig. 4:**
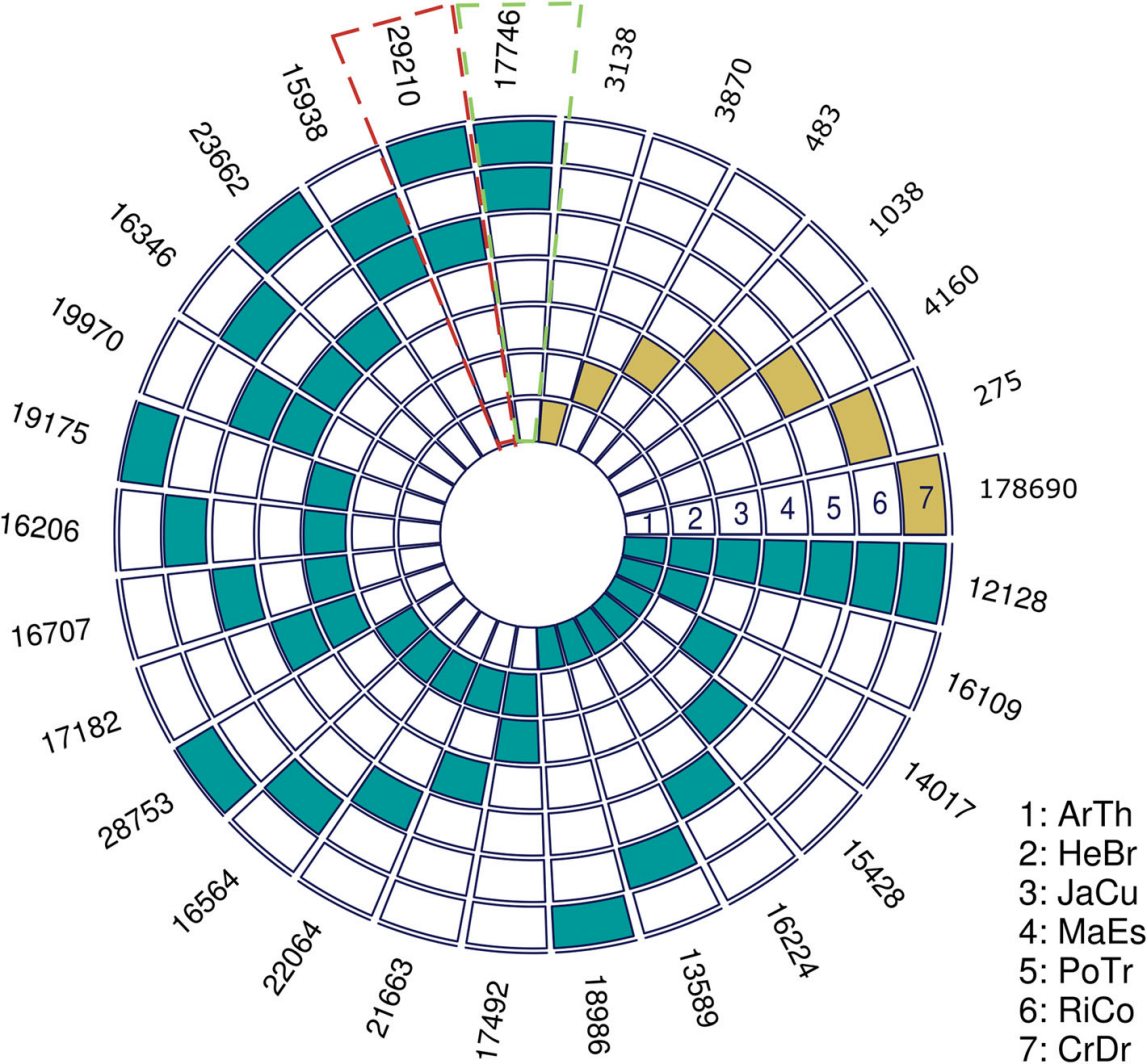
Number shared orthologous and unique genes identified between compared plant species. Each concentric circle corresponds to one species [(1) ArTh, *Arabidopsis thaliana*; (2) HeBr, *Hevea brasiliensis*; (3) JaCr, *Jatropha curcas*; (4) MaEs, *Manihot esculenta*; (5) PoTr, *Populus trichocarpa*; (6) RiCo, *Ricinus communis*; and (7) CrDr, *Croton draco*]. Blue blocks mark the intersection by pairs (orthologs between two species), yellow blocks reference unique genes for each species (species-specific genes). The number of shared orthologs is shown next to the last concentric circle. *C. draco* shares the largest number of orthologs with *P. trichocarpa* (box highlighted with dark blue dotted line) and the least with *R. communis* (box highlighted pink dotted line). To generate this figure and visualize the multi-species intersections, we used the R package SuperExactTest [42]

Using the OrhoMCL algorithm [41] we identified orthologous genes in all the reference species used in the present study. A total of 281,493 proteins (127,919 from *C. draco*; 22,463 from *A. thaliana*; 33,563 from *H. brasiliensis*; 19,865 from *J. curcas*; 25,361 from *M. esculenta*; 34,145 from *P. trichocarpa*; 18,177 from *R. communis*) were assigned to 41,392 orthogroups (Additional file 8: Table S7). Among these, 9717 orthogroups were shared among all seven-reference species; 1326 orthogroups contained proteins from plant species in the Malpighiales order, but not in *Arabidopsis* and 525 orthogroups contained putative one-to-one single copy genes (Fig. 4 and Additional file 7: Table S6).

### Identification and expression analysis of candidate genes involved in magnoflorine biosynthesis

The aporphine alkaloid magnoflorine is a probable biosynthetic precursor of taspine [24]. In order to identify the principal enzymes involved in magnoflorine biosynthesis, we performed an extensive literature review. The current understanding of magnoflorine biosynthesis comes mainly from a study in *Coptis japonica* [43], in which a C-C phenol coupling of (*S*)-reticuline by a cytochrome P450 (CYP80G2) appears to responsible for producing the aporphine scaffold in (*S*)-corytuberine, which can subsequently N-methylated to produce magnoflorine. Alternatively, in opium poppy (*Papaver somniferum*) the N-methylation of (*S*)-reticuline yields the quaternary alkaloid tembetarine, which may also serve as a substrate for a CYP80G2-like enzyme that forms magnoflorine via an alternative route [44] (Additional file 7: Figure S5). The C-C phenol-coupling reaction involved in magnoflorine biosynthesis is analogous to the conversion of (R)-reticuline to salutaridine required in morphine biosynthesis [45, 46]. The biosynthesis of morphine and related alkaloids in *P. somniferum* and related species occurs via a multistep pathway beginning with L-tyrosine [45], which is converted to (S)-reticuline in several steps. (S)-reticuline is then converted to its stereoisomer (R)-reticuline, a unique case among the many benzylisoquinoline pathways that are known [47]. In subsequent steps (R)-reticuline is converted to morphine (Additional file 7: Figure S5). Most of the enzymes involved in the major biosynthetic steps from tyrosine to morphine [48] and/or magnoflorine [44] have been functionally characterised in *P. somniferum* and some have been identified in other plants including *Coptis japonica* [43, 49], *Eschscholzia California* [50], *Podophyllum* spp. [51] and *Thalictrum flavum* [52].

**Fig. 5:**
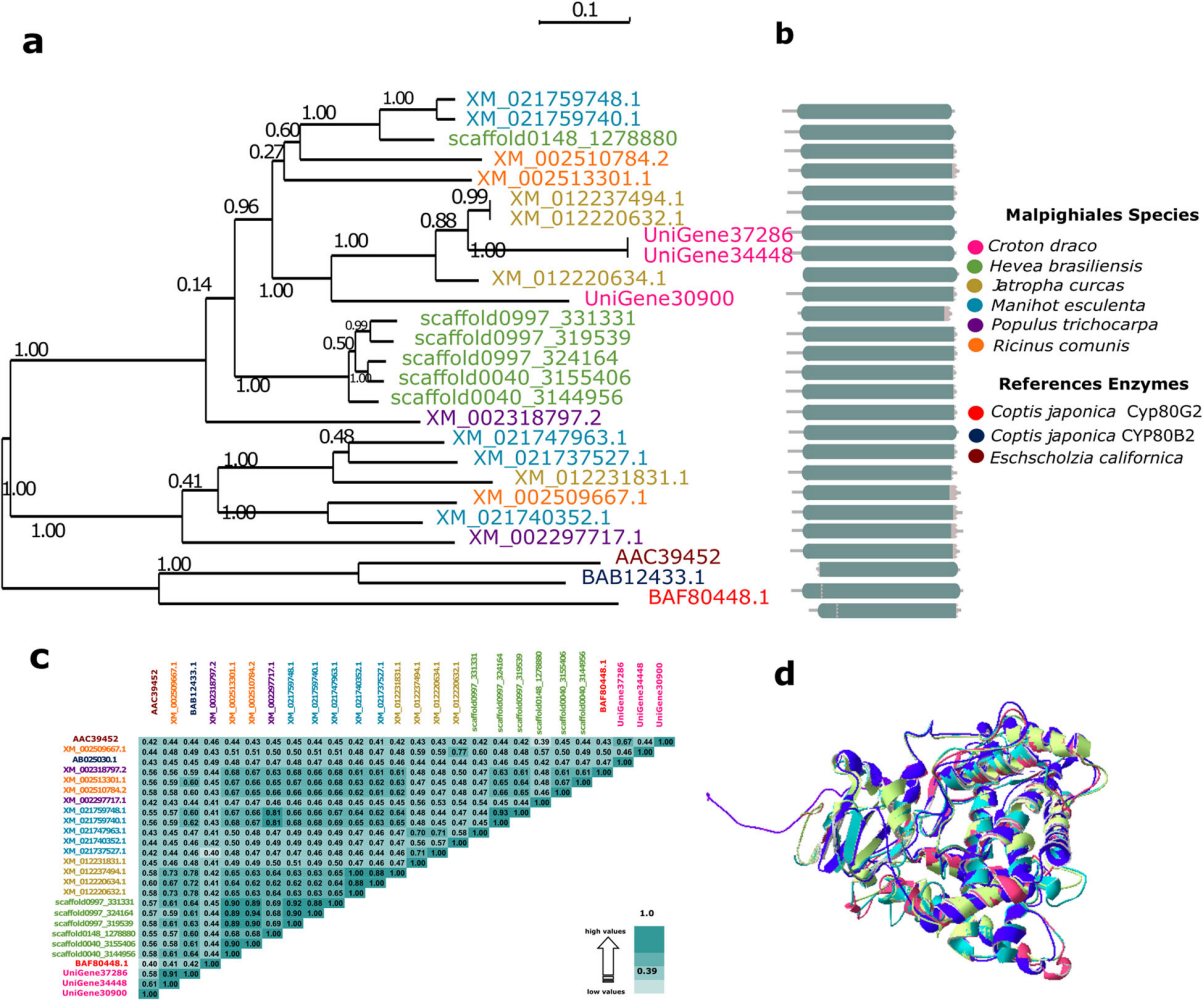
Evolutionary relationships among the proteins grouped as orthologs of the (S)-corytuberine synthase (CTS; CYP80G2, reference protein with accession number: AB288053.1). **a** Maximum likelihood phylogenetic tree depicting the relationship between the proteins grouped as orthologous from the different plant species that were compared. **b** Primary protein structures in which the Cytochrome p450 (CYP450) conserved domain (PF00067) are represented by cyan-grey boxes. **c** Matrix of the percentage identities between the aligned CTS orthologs. **d** Structural model superposition of CTS proteins including those from the *C. draco* species [UniGene30900 (green), UniGene34448 (blue) and UniGene37286 (pink)], and the CTS reference protein from *Coptis japonica* (purple). See methods for more details

Based on published information and using the OrthoMCL software in a combination with phylogenetic analyses, we identified *C. draco* candidate genes that are likely to be involved in magnoflorine biosynthesis (Additional file 7: Figure S5 and Table 1). Orthogroups that contained some of the reference enzymes [tyrosine decarboxylase (TYDC), (S)-norcoclaurine synthase (NCS), (S)-norcoclaurine 6-O-methyltransferase (6OMT), (S)-coclaurine N-methyltransferase (CNMT), (S)-N-methylcoclaurine 3′-hydroxylase (NMCH; CYP82B subfamily), (S)3′-hydroxy N-methylcoclaurine 4′-O-methyltransferase (4’OMT), (S)-corytuberine synthase (CTS, CYP80G2) and reticuline N-methyltransferase (RNMT)] were extracted from the OrthoMCL output (Additional file 7: Table S6) in order to identify the sequences of the orthologs present in some of the reference species used in the present study, including the *C. draco* unigenes. We then performed the alignments of all identified proteins in order to generate the corresponding phylogenetic tree and confirm the presence of well-conserved domains that are distinctive from each type of enzyme. The alignments of the proteins included in the orthogroups containing some of the reference proteins were curated manually (we only considered those proteins that represented at least 70% of the length of the complete proteins). Figure 5 shown the Maximum Likelihood (ML) phylogenetic tree, a graphical representation in which conserved domains of the aligned ortholog proteins are represented, the identities matrix and finally (only for this case used as an example), structural model superposition of *C. draco* proteins and their orthologs used as a reference for the (S)-corytuberine synthase enzyme (CTS).

**Table 1:** Plant enzymes functionally characterized and involved in magnoflorine biosynthesis

Enzyme		Source plants	Reference*
Acronyms and/or abbreviations	Name [GeneBank accession number]		
TYDC	tyrosine decarboxylase [AAA62346.1|AAA62347.1]	*Papaver somniferum*	[53]
NCS	(S)-norcoclaurine synthase [BAF45337.1|AAR22502.1]	*Coptis japonica* *Thalictrum flavum*	[54] [55]
6OMT	(S)-norcoclaurine 6′-O-methyltransferase [AAQ01669]	*Papaver somniferum*	[56]
CNMT	(S)-coclaurine N-methyltransferase [BAB71802.1]	*Coptis japonica*	[57]
NMCH (CYP82B subfamily)	(S)-N-methylcoclaurine 3′-hydroxylase [AC39454.1]	*Eschscholzia california*	[50]
4OMT	(S)3′-hydroxy N-methylcoclaurine 4′-O-methyltransferase [AAP45313.1|BAB08005.1]	*Coptis japonica*	[58]
CTS (CYP80G2 subfamily)	(S)-corytuberine synthase [BAF80448.1]	*Coptis japonica*	[43]
RNMT	reticuline N-methyltransferase [AOR51553.1|AOR51552.1]	*Papaver somniferum*	[44]

*References mainly correspond to recent reports in which the activity of these enzymes was proved. Additional studies confirm the presence of most of these enzymes in species such as *P. somniferum* and *C. japonica*

**Fig. 6:**
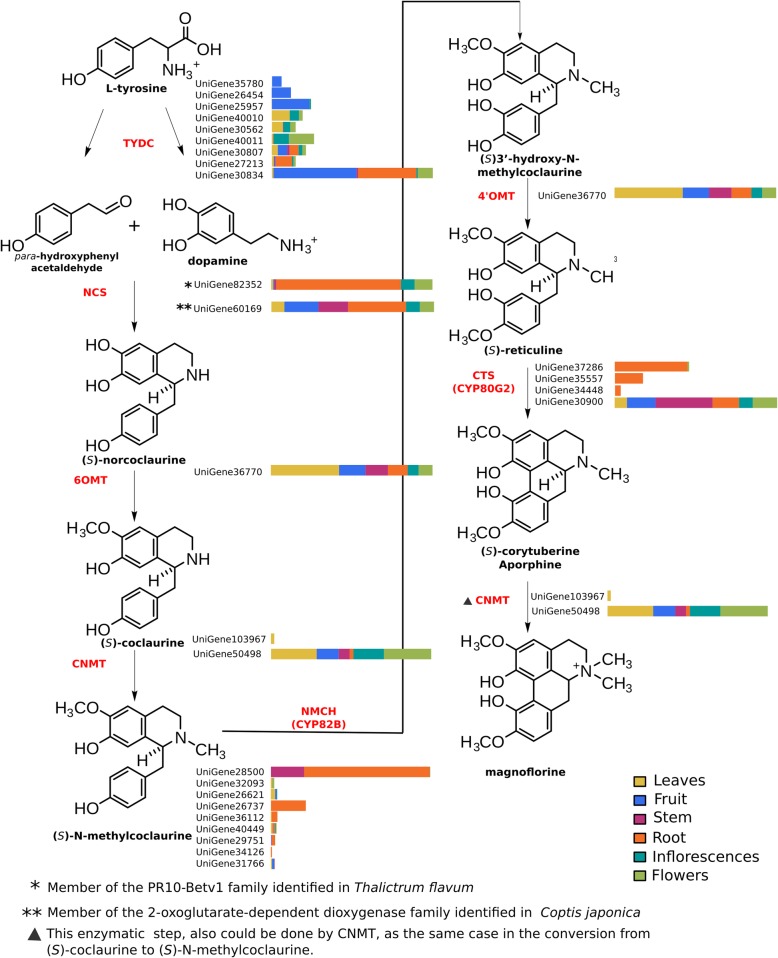
Schematic representation of magnoflorine biosynthetic pathway and the expression profile of those unigenes of the *C. draco* species which coding for the enzymes involved. In the figure, the molecular structure of each proposed intermediate is represented, followed by the enzymes (acronyms shown in red) and their corresponding orthologs identified in the *C. draco* transcriptome. Expression profiles representing the abundance of the transcripts (FPKM) in each organ analysed are also shown for each of the *C. draco* unigenes identified as orthologs of the reference enzymes. Acronyms: TYDC (tyrosine decarboxylase); NCS ((S)-norcoclaurine synthase); 6OMT ((S)-norcoclaurine 6-O-methyltransferase); CNMT ((S)-coclaurine N-methyltransferase); NMCH ((S)-N-methylcoclaurine 3′-hydroxylase, CYP82B subfamily); 4’OMT ((S)3′-hydroxy N-methylcoclaurine 4′-O-methyltransferase); CTS ((S)-corytuberine synthase, CYP80G2)

According to the identity matrices (Additional file 8: Tables S7-S13), similarities among sets of orthologous enzymes ranged from 23 to 70%. In the cases in which orthologs were identified in all the reference species included in the analysis, clades in the phylogenetic trees were congruent with those of the species tree. This was to be expected since according to the phylogenetic definition, two homologous genes are orthologous if they diverged from a shared speciation event. Therefore, the phylogenetic tree of a set of orthologs (a set of genes in which any pair is orthologous) is bound to have the same topology as the corresponding species tree. All unigenes identified after the analyses described above were considered as the primary candidates *C. draco* genes involved in the proposed biosynthetic pathway of magnoflorine, (Fig. 6), the primary precursor of taspine.

Magnoflorine biosynthesis begins with a condensation reaction of dopamine and para-hydroxyphenylacetaldehyde, both of which are derived from L-tyrosine. The norcoclaurine synthase enzyme catalyses this reaction but, interestingly, two enzymes belonging to two different protein families are capable of catalysing the reaction, both of which were included in our list of reference proteins. One of these enzymes identified in *Thalictrum flavum* [55] is induced in defence responses and belongs to the PR10/Betv1 family of proteins. The other enzyme, identified in *Coptis japonica*, is a member of the 2-oxoglutarate-dependent dioxygenase family [54]. With only one exception (reticuline N-methyltransferase, RNMT) orthologs from all reference enzymes involved in magnoflorine synthesis were identified in the *C. drado* transcriptome. Moreover, the enzymes (S)-norcoclaurine 6-O-methyltransferase and (S)3′-hydroxy N-methylcoclaurine 4′-O-methyltransferase (6OMT and 4’OMT, respectively), were grouped in the same orthogroup with a unique *C. draco* unigene (Additional file 7: Table S6).

The absence of an ortholog to the RNMT enzyme strongly suggests that the route to magnoflorine from (s)-reticuline is via the synthesis of (S)-corytuberine as an intermediary in *C. draco* (Fig. 6, see also Additional file 7: Figure S5). In this pathway, (s)-reticuline is the C8-C2’ substrate coupling which yields (S)-corytuberine, a step catalysed by the activity of the enzyme (S)-corytuberine synthase (CTS, CYP80G2), which is followed by the N-methylation required for magnoflorine synthesis. This final step could be catalysed by the (S)-coclaurine N-methyltransferase (CNMT), an enzyme which in the *C. japonica* species can accept (S)-corytuberine as a minor substrate [49]. Furthermore, biosynthesis of the central isoquinoline alkaloid intermediate (S)-reticuline requires the 6OMT and 4’OMT enzymes [56–58]. Even when these enzymes share a high degree of sequence identity, the presence of both enzymes in several plant species suggests that they may have strict substrate specificity and may be able to tolerate only minor variation in the presumed substrates in vivo. However, there is evidence that some O-methyltransferases are surprisingly permissive in the range of substrates that they can methylate [59, 60]. Therefore, it is possible that in *C. draco* a unique O-methyltransferase can methylate distinct substrates such are those recognized by the 6OMT and 4’OMT enzymes in other species (Fig. 6, see also Additional file 7: Figure S5).

In addition to their identification based on RNA-seq data, we analysed the expression patterns of all candidate genes likely to be involved in the magnoflorine biosynthetic pathway (Fig. 6). This analysis revealed that these genes are ubiquitous, as indicated by the abundance of their transcripts in all the plant organs evaluated. Also, even when their expression levels varied in different organs, none of the genes was expressed differentially or expressed in an organ-specific manner. This result was predictable since magnoflorine was present in each of the sampled organs except in the latex sample extracted from the bark of *C. draco*.

### Magnoflorine as the probable biosynthetic precursor of taspine

**Fig. 7:**
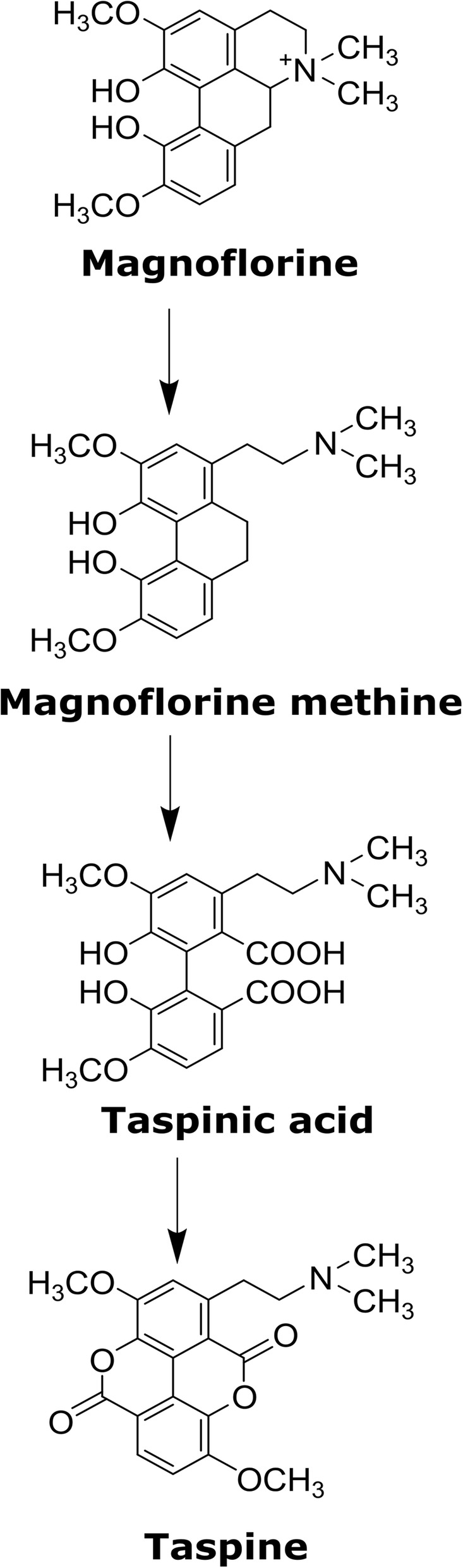
The biosynthetic pathway from magnoflorine to taspine in *C. draco*. The main intermediates in the pathway are shown based on the previously proposed pathway [24]

According to the biosynthetic pathway proposed by Shamma and Moniot [24], several enzymatic reactions are required to achieve the conversion of magnoflorine to taspine (Fig. 7). However, bioassays involving these enzymes have yet to be performed. Similarly, no enzymes capable of catalysing these reactions have been functionally characterized, so that the identification of candidate genes involved in this pathway through the search for orthologs is not a feasible strategy. Some of the intermediates suggested as the precursors in the taspine biosynthetic pathway, as well as some of those involved in magnoflorine biosynthesis, were tentatively identified based on the LC-HRMS results, comparison of their spectrometric fingerprints with those reported in METLIN-Scripps databases, considering errors less than 3.0 ppm (Additional file 4: Figure S6 and Additional file 9: Table S14). Metabolomic analysis performed with HRMS are frequently used to identify precursors involved in biosynthetic pathways [61, 62] and the integration of metabolomics and next-generation sequencing data to elucidate pathways in plants had contributed to new important discoveries [63].

(S)-Reticuline and (S)-corytuberine, which are immediate precursors of magnoflorine, were present at increasing concentrations in roots followed by stems, leaves, and flowers. Interestingly, (S)-reticuline was not detected in roots. In contrast, magnoflorine was most abundant in roots and flowers rather than stems and leaves (Additional file 4: Figure S6 and Additional file 9: Table S14). Interestingly, tembetarine, one of the intermediates that could be a precursor in some plant species in which magnoflorine is synthetized, was not detected in any of the organs analysed. The absence of tembetarine, which is consistent with the absence of an ortholog of the RNMT enzyme and amounts of (S)-reticuline and (S)-corytuberine identified by LC-HRMS in the organs sampled, provide additional evidence for the magnoflorine biosynthetic pathway proposed for *C. draco*. Regarding taspine and their intermediaries, magnoflorine methine and taspinic acid, a similar profile was observed for each of these metabolites in each of the organs sampled (Additional file 4: Figure S6 and Additional file 9: Table S14). Both magnoflorine and taspine were consistently the most abundant metabolites detected in the organs analysed. Based on these results our data provide robust evidence for the proposed pathways involved in magnoflorine and taspine biosynthesis in the *C. draco* species.

### Quantification and identity confirmation of taspine and magnoflorine

**Fig. 8:**
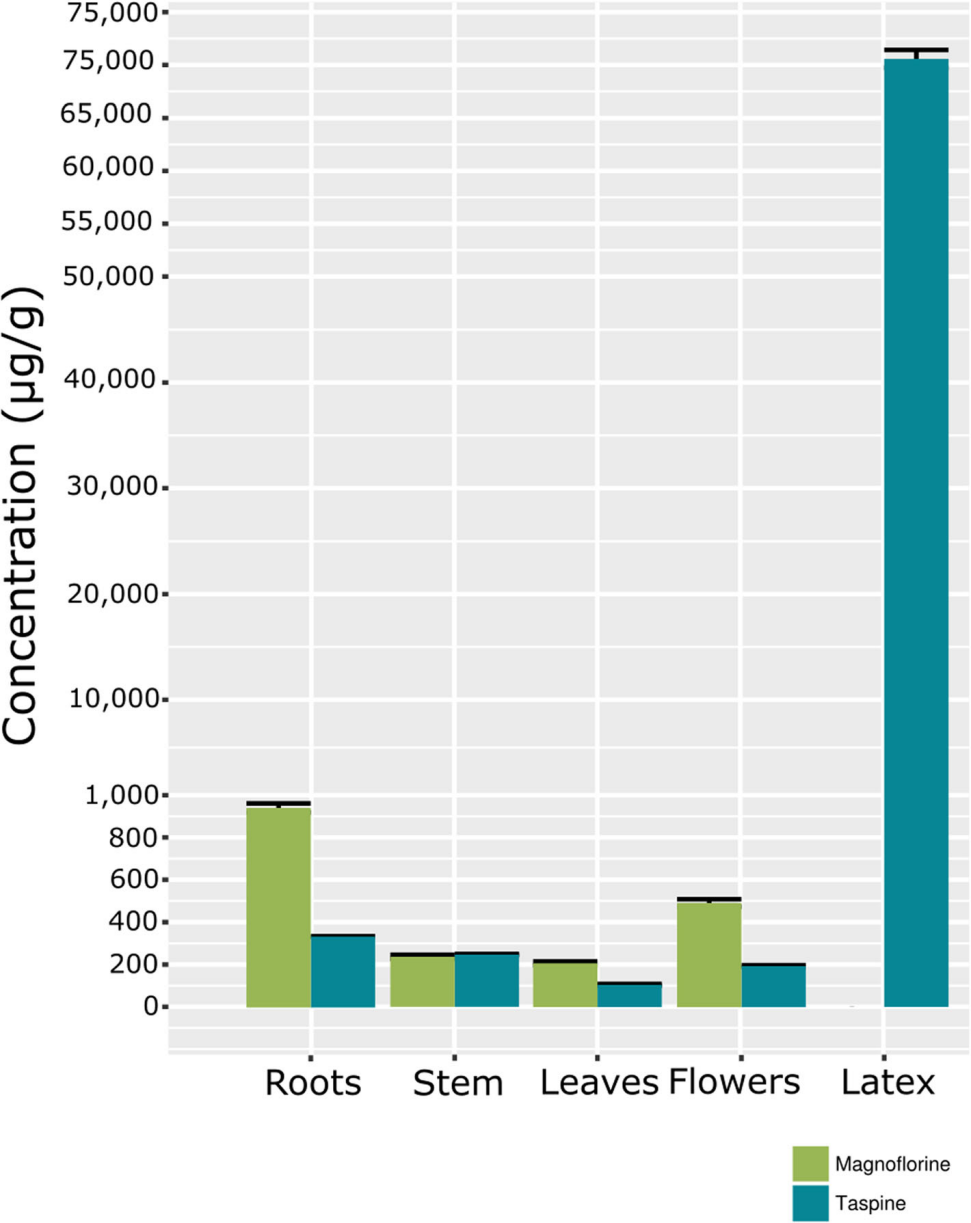
Magnoflorine and taspine content (μg/g) it the organs of the plant (roots, stem, leaves and flowers) and in latex harvested from the bark of the main trunk, quantified by LC-MS. Bars represent the mean values ± SD of three biological replicates

The amounts of magnoflorine and taspine metabolites were quantified by spectral matching with the commercial standards in LC-MS assays in the different organs and latex. The patterns of distribution of both magnoflorine and taspine were the same with respect to those observed in the LC-HRMS analyses (slightly more abundant in flowers and roots with respect to stems and leaves; Fig. 8). Taspine, which is present in some other species of the *Croton* genus [18], was also found in abundance in the latex of *C. draco* (around 7% of dry weight). Taspine was significantly more abundant in the latex from the bark of the trunk in comparison with the other organs sampled. Taspine accumulates only in laticifer cells and this metabolite represents a considerable fraction of the total dry weight of latex. Interestingly, magnoflorine was not detected (Fig. 8). This is consistent with a previous report that detected magnoflorine in some tissues but not in the latex of the *C. lechleri* [18].

### Abundance of laticifer cells in the organs of *Croton draco*

**Fig. 9:**
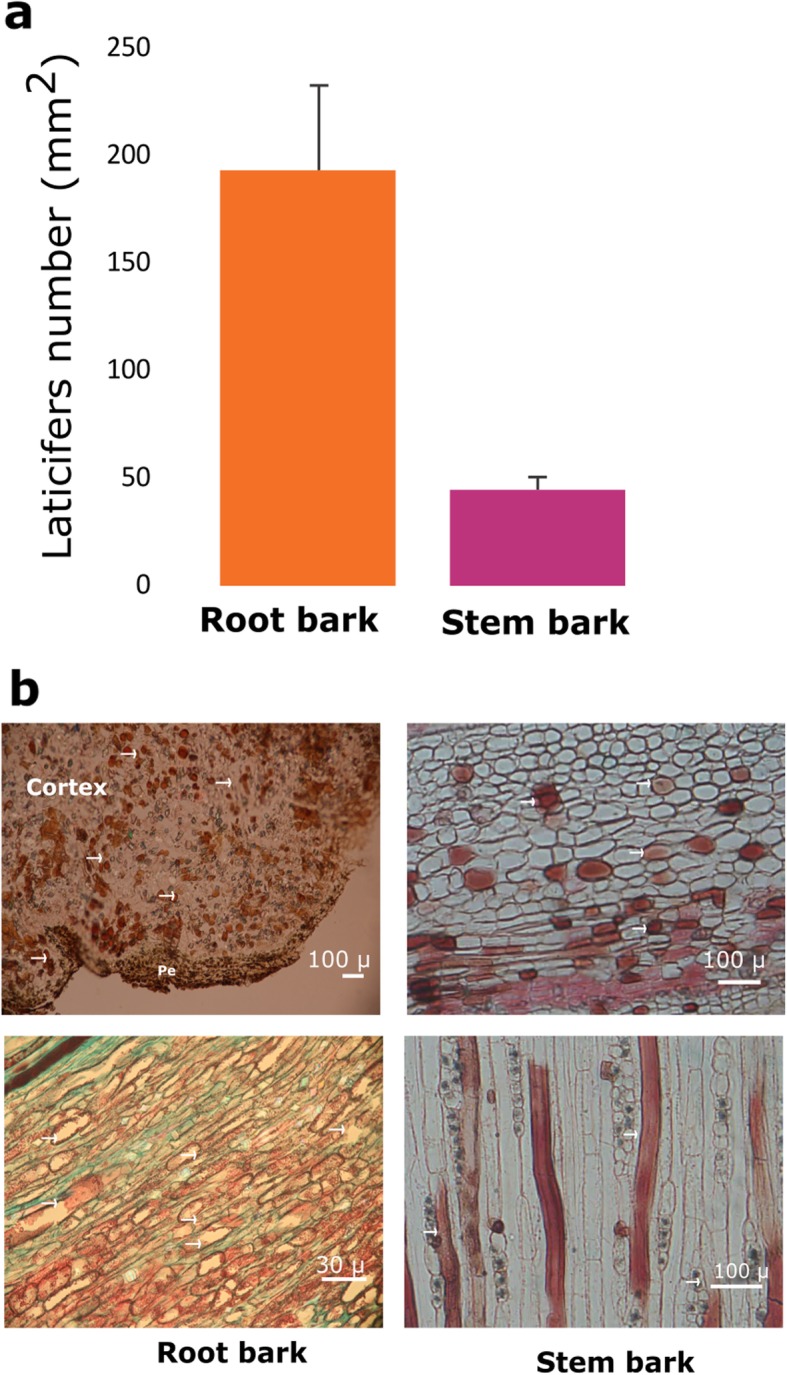
Laticifer cell abundance in the roots and the bark of stems of *C. draco*. Panel (**a**) shows the density of laticifer cells per mm^2^. Panel (**b**) shows photomicrographs of transverse sections (up) and tangential sections (below) of the cortical region of the bark of the roots (left) and stem (right) stained with safranin-fast green, and safranin, respectively. Peridermis (Pe) and the laticifer cells are shown with arrows (→) in the photomicrographs of transverse sections

To determine whether the abundance of *C. draco* laticifer cells was related to the concentration of secondary metabolites such as taspine, we quantified the laticifer cell densities in the cortical region of the bark of the stem and roots. We observed that taspine was more abundant in the roots than in the stem, and it is the cortical region of the roots that have the highest density of laticifer cells (Fig. 9).

## Discussion

In latex-bearing plants, laticifer cells are present mainly in the leaves and/or stems, although in other plant species, laticifer cells can found it in other organs and tissues [64]. In *Croton* species, laticifer cells are mainly located in the bark phloem, and their abundance is dependent on plant age, tree location and the environment [4, 65]. Laticifers are less abundant in old than in young stems [66]; the branches have higher laticifer densities than the stem and trees of the tropical rainforest have more laticifers than those of semi-deciduous tropical forest [1]. As in other species in which specific metabolite accumulation is related to the abundance of laticifer cells [67], in *C. draco* the accumulation of taspine seems to correlate with the abundance of laticifer cells. In this study, we proved that magnoflorine, a precursor for taspine biosynthesis, was present in the methanolic fraction obtained from several organs. However, this alkaloid was not present in latex harvested from the bark of the main trunk. These results suggest that, as occurs in *Podophyllum* species, some of the genes involved in magnoflorine biosynthesis could be transcribed in specific cell types [51]. It is probable that in *Croton* species, following synthesis of magnoflorine in a specific cell type, this compound has to be translocated to the laticifer cells in order to be converted and stored as taspine. We hypothesised that magnoflorine could not be detected in latex because it is a short-lived metabolite and a highly reactive intermediary which quickly gives rise to the production of taspine and their precursors.

In addition to provide evidence of the presence of magnoflorine and taspine in four distinct organs of the plant, this study used transcriptomic data and phytochemical analyses to propose a biosynthetic pathway for these two metabolites. Key findings, such as the absence of an RNMT enzyme, suggest that in *C. draco* CNMT might act as a bifunctional enzyme capable of recognizing two different substrates: (S)-coclaurine and (S)-corytuberine. Also, considering that a single *C. draco* unigene is an ortholog to both 6OMT and 4’OMT enzymes, it seems that additional steps in magnoflorine synthesis require another O-methyltransferase capable of recognizing two different substrates.

As expected, the transcriptional atlas of *C. draco* unigenes reveals that plant organs differ in morphology and in their functional roles. We observe that the expression profiles of each of the *C. draco* unigenes considered as prime candidates involved in the distinct enzymatic steps required to convert tyrosine to magnoflorine, were not expressed in an organ-specific manner and some of them are more abundant in some organs than in others.

This apparent expression dynamism is probably related to the translocation of some metabolites or intermediaries from the organ where they are produced to another organ in which they preferentially accumulate. Some transporters responsible for alkaloid or other secondary metabolite translocation have been described [68–70] and shown to function in experimental studies [70, 71]. For example, the alkaloids that exhibit strong biological activity as plant defence mechanisms against pathogens and herbivores, sometimes need to be translocated via the xylem from tissues where they are synthesized to the accumulation sites (e.g., the vacuoles of cells in a different organ). This active transport may allow the plants to cope with the toxicity of these compounds. A canonical example of this phenomenon is nicotine, the major alkaloid of the *Nicotiana* species, which is translocated via the xylem from the root tissues, where it is synthesised, to the accumulation sites in the vacuoles of leaves [72].

Considering that both magnoflorine and taspine are classified as aporphine alkaloids and they have been extensively studied for their toxicity in distinct cancer cell lines [21, 73], it is likely that their biosynthesis involves a complex process which requires distinct organs, different cell types and active transport of some intermediates that have to be translocated.

As a single cell type can harbour thousands of metabolites, for many of which neither their structure, function nor their pharmacological potential are known, the medicinal plants could be considered both as a tremendous challenge to plant chemists but also an unlimited catalogue of bioactive compounds, enzymes, transporters, or regulators that could be used in synthetic biology programs and for medical and veterinary applications. Fortunately, Next Generation Sequences technologies and accurate mass spectrometry-based techniques, along with nuclear magnetic resonance spectroscopy, can be employed to sequence “non-model” plant transcriptomes and identify enzymes involved in the biosynthesis of these compounds. Integrating these studies with metabolomics based on mass spectrometry, will allow us to identify novel compounds and elucidate their biosynthetic pathways. In the present study we employed an integrative approach combining multi-omics tools to produce the first evidence on the enzymes involved in magnoflorine and taspine synthesis in *C. draco*.

## Conclusions

Identification of genes involved in aporphine alkaloids biosynthesis, such as magnoflorine and taspine, will contribute to future functional studies in the plant and provide a basis for improving production levels in plants or even in microbial hosts by metabolic engineering. In our data set, we not only identified transcripts that encode for enzymes involved in the metabolic pathways of these metabolites with anticancer and plant defence properties, but we also show evidence by the identification of some intermediates, the pathway that has a place in *C. draco* to produce these important compounds. Further, we annotated many genes involved in the various metabolic pathways. The dataset of assembled *C. draco* unigenes presented in this study is the first set of omics data available for the species and will provide the foundation for other functional and comparative genomic studies.

## Methods

### Biological material

All samples used in this study were collected from well-established three-year-old trees. These trees were considered to be clones (at least in the aerial part) since they were propagated from branches of a single individual that were grafted on three-month-old rootstocks grown from seeds. The *C. draco* plantation is located in the “El Palmar” Experimental Centre of the National Institute for Forestry, Agriculture and Livestock Research (INIFAP), in the municipality of Tezonapa, Veracruz, Mexico. A representative specimen was deposited in the XAL Herbarium (INECOL A.C., Xalapa, Mexico) with accession number FRF-316. The samples collected to be used in the present study were leaves, stems and roots from a single tree. Fruits, inflorescences and flowers were also sampled, the latter two organs with and without floral stem, respectively. All samples were immediately frozen in liquid nitrogen, taken to the laboratory and then stored at -80 °C until use.

While it is true that biological replicates are recommended in RNA-seq experiment, this is especially important when samples come from individuals which, despite to be part of the same population, could be genetically different. However, samples used in the present study come from clonal plants which were growing under the same conditions; therefore, considerable genetic variation is not expected to be in the case of the clones used. The main objective of our sampling was mainly to obtain a transcriptional atlas from different organs, in which as many genes as possible were included. In addition, some recently published studies evaluating different treatments or conditions, do not show independently-sequencing biological replicates; even though, they have shown confident and conclusive results [74]. Due to the above described, samples from distinct organs analysed were harvested from five different trees, then were pooled and considered as unique samples at the time of RNA isolation and library preparation.

### RNA isolation, library preparation, and sequencing

The harvested samples were pulverized, and total RNA was isolated from 200 mg of homogenized tissue. RNA was isolated according to a previously described method for plants with substantial amounts of polysaccharides and polyphenolic compounds [75]. RNA integrity was evaluated by chipbased capillary gel electrophoresis using a Bioanalyzer system (Agilent Technologies). RNA concentration was determined by absorbance at 260 nm using a UV spectrophotometer (BioSpectrometer Eppendorf). A sample of 500 ng of RNA was used as input material for the cDNA library preparation. One library for each plant organ was prepared using standard TruSeq RNA Sample Preparation Kit (Illumina) and sequenced using the Illumina NextSeq500 platform to obtain 150-bp paired-end reads. Files containing sequence reads and quality scores were deposited in the Short Read Archive (SRA) of the National Center for Biotechnology Information (NCBI). Accession number SRP156345.

### Quality control of sequenced reads and de novo transcriptome assembly

Before assembly, the removal of low-quality paired-end-reads was carried out using the qualityControl.py script from Next-Generation Sequencing (NGS) toolkits (https://github.com/Czh3/NGSTools/blob/master/qualityControl.py). The stringency parameters used to select High Quality (HQ) paired-end reads were: -q 30 (Minimum quality score to keep), -p 90 (Minimum percentage of bases that must have [-q] quality) and -a 30 (the minimum average quality in the selected paired-end reads). Overlapping pairs of sequencing reads were merged using the SeqPrep program (https://github.com/jstjohn/SeqPrep), which removes barcodes and contaminating adapter sequences and avoids the generation of orphan reads. Transcriptome de novo assembly was performed including adapters-free HQ paired-end reads and those more extended sequences resulting from merging reads (R1 and R2) through their overlapping regions. Trinity assembler (v2.4.0) [76] with default parameters was used for this purpose in where all datasets generated (libraries from leaves, stems, roots, fruits, inflorescences and flowers) were combined. In the resulting contigs (unigenes), regions such as poly A/T tails, ends rich in Ns (undetermined bases), and low complexity sequences were trimmed using SeqClean software (http://compbio.dfci.harvard.edu/tgi/), whereas environmentally-derived contaminant sequences were removed using DeconSeq software [77]. Considering that DeconSeq requires a pre-built database of potential contaminant sequences, we generated this from repetitive DNA elements (RepBase; http://www.girinst.org/repbase/) and all transcripts derived from gene models predicted in the sequenced genomes and available in GenBank for insects, bacteria and fungi (https://www.ncbi.nlm.nih.gov/genome/). The final unigenes from *C. draco* were generated from contamination-free contigs with a minimum length of 200 bp.

### Identification and annotation of protein-coding regions

To generate an accurate and non-redundant dataset from the *C. draco* transcriptome, sequences corresponding to the coding regions were extracted from the full-length unigenes. Considering that insertions and deletions cause loss of open reading frame and both are common errors in sequences assembled from next-generation techniques [78], likely frameshift errors in coding sequences were corrected before translating the corresponding proteins. AlignWise pipeline [27] was used for this purpose. Once coding sequences (CDS) had been identified and corrected, BLASTClust program (ftp://ftp.ncbi.nih.gov/blast/documents/blastclust.html/) was used to create a non-redundant set of sequences. Unigenes in which CDS were 90% identical over at least 90% of the length of another CDS, were removed to avoid redundancies. Additionally, CDS that resulted in sequences no longer than 75 amino acids were removed, as the likelihood of finding similarity to previously described proteins is highly dependent on the length of the query sequence [28]. As part of the annotation process, BLAST searches using the single-directional best hit method (e-value 10^− 05^) were performed to identify in each set of reference proteins, those homologous to the proteins translated from *C. draco* unigenes. As reference proteins, we used the proteome derived from the predicted gene models of several complete plant genomes. With only one exception (*Arabidopsis thaliana*; http://www.arabidopsis.org/), all the reference proteomes were those of angiosperms in the order Malpighiales [*Hevea brasiliensis* [79], *Jatropha curcas* [80], *Ricinus communis* [81], *Populus trichocarpa* [82] and *Manihot esculenta* [83]]. Gene Ontology (GO) annotation was assigned based on similarity to *A. thaliana* proteomic sequences. KEGG pathways and enzyme codes were assigned to *C. draco* unigenes using the online KEGG Automatic Annotation Server (KAAS) at http://www.genome.jp/tools/kaas/ of the KEGG (Kyoto Encyclopedia of Genes and Genomes) database. Finally, to screen and identify conserved protein domains, we used the HMMER software package (v3.2.1) ([84]; http://hmmer.org/) and Pfam database (http://pfam.sanger.ac.uk) with an inclusion threshold of 0.0001.

### Ortholog groups identification

Orthologous and paralogous genes were identified via the Markov Cluster Algorithm (MCL) with an inflation value of 1.5 [85]. OrthoMCL program [41] was used for this purpose. OrthoMCL [41] is a graph-clustering algorithm designed to identify homologous proteins based on sequence similarity and distinguish orthologous from paralogous relationships without computationally intensive phylogenetic analysis. For the clustering process, we considered a minimum input length of 30 amino acids in all compared proteins, as well as a threshold value of 10^− 10^ in the BLAST step. This stringent cut-off value was chosen to avoid false positive results. Splicing variants were removed from each of the proteomes compared and the longest predicted isoform was selected as the representative transcript for each gene. Proteomes included in orthology analyses (all from the species mentioned in the previous section in the order Malpighiales and *A. thaliana*) were complemented by adding the sequences of some proteins from additional plant species. These enzymes were included because all of them had been functionally characterised and were known to be involved in magnoflorine biosynthesis (reference enzymes were described in the text).

### Identification of differentially expressed genes (a transcriptome atlas)

To generate a comprehensive atlas of global transcription profiles of the different plant organs, the high-quality R1-R2 read pairs from each sample were aligned to the annotated and non-redundant *C. draco* unigenes set considered as the reference transcriptome. To minimise bias and avoid false-positive results in differential expression analysis, around 7 million randomly-selected reads from each library were used in the mapping process. For this, we used RSEM (RNA-Seq by Expectation-Maximization) pipeline [86], which handles and directly executes Bowtie2 [87] using predefined and optimized parameters in the mapping process and calculates maximum likelihood abundance estimates, as well as posterior mean estimates and 95% credibility intervals for genes, based on the expected read counts. RSEM also computed normalised expression values as Fragments Per Kilobase per Million (FPKM) and Transcripts Per Million (TPM). Based on this information, an expression matrix was constructed from rows and columns that denoted the unigenes and their corresponding transcript levels (represented by the expected read counts, and TPM and FPKM values) from each organ sampled. A threshold of FPKM ≥3 was used to distinguish expressed genes in at least one of the organs sampled from those whose calculated values can be considered as background-noise [88]. To compare expression levels of the *C. draco* unigenes between the analysed organs, Perl scripts included in the Trinity software package [76] were used to perform a normalisation process using the TMM (weighted Trimmed Mean of M-values) method [89, 90] and the subsequent identification of differentially expressed genes by using the EdgeR Bioconductor package [89]. The differential gene expression analysis focused on identifying those transcripts (unigenes) that were specific or preferentially expressed in each organ and distinguish from those that can be considered as ubiquitous. For organ-wise paired comparisons, the dispersions were estimated and then used for logFC (log_2_ fold change) calculation. Also, a false discovery rate (FDR) adjusted *P*-values were calculated by using the Benjamini-Hochberg approach [91]. FDR ≤ 0.001 and logFC ≥1 were used as thresholds to judge the significance of differences in transcript abundance of the same *C. draco* unigene between two distinct organs. FPKM values estimated for each unigene were used to compare expression levels among samples. Finally, to identify overrepresented biological functions in the analysed organs, a gene ontology (GO) functional enrichment analysis was performed for the differentially expressed genes using BiNGO (https://www.psb.ugent.be/cbd/papers/BiNGO/Home.html). The analysis was performed with a *P*-value < 0.05 based on a hyper-geometric test and the Benjamini-Hochberg FDR correction.

### Quantitative PCR analysis

The validation of RNA-seq data was carried out using reverse transcriptase quantitative PCR (RT-qPCR) analysis. The RNA used for qPCR was the same as that used for RNA-seq. cDNA templates for qPCR were prepared from isolated RNAs using SuperScript III reverse transcriptase (Invitrogen). Primers for each gene were designed using Primer3 v.0.4.0 (http://bioinfo.ut.ee/primer3-0.4.0/), to produce the primer sequences shown in Additional file 9: Table S15. RT-qPCR was conducted in a STRATAGEN MX3005P (Agilent Technologies) real-time thermal cycler using SYBR Green® PCR Master Mix (Life Technologies). The amplification procedure comprised an initial denaturation step at 95 °C for 30 s, 40 cycles at 95 °C for 5 s, 60 °C for 30 s, and 72 °C for 30 s. PCR specificity was assessed by melting curve analysis. The expression of *actin-2* (AT5G09810|UniGene122200) was used as an internal control. It is worth mentioning that it has been previously reported that for some plant species, *actin-2*, is one of the best housekeeping genes when different tissues and/or developmental stages, are compared [92]. Its stability in *C. draco* was confirmed after comparing the calculated FPKM values across all organs sampled was confirmed (8.685 ± 1.337 SE). Each reaction was performed in triplicate for the selected *C. draco* unigenes, and the fluorescence intensities of each gene, as measured by cycle threshold (Ct) values, were compared using the 2^(−∆∆Ct)^ method [93].

### Phylogenetic analyses

Proteins from all plant species included in our analyses were grouped as orthologs of the reference enzymes and were processed with SeaView [94]. First, using the MUSCLE v3.8.31 program [95], amino acid sequences were used to guide the alignment of their corresponding coding sequences. Phylogenetic trees were generated in a maximum likelihood framework (ML) using the general time reversible (GTR) substitution model. PhyML v3.0 software [96] was used for this purpose. In addition, equilibrium frequencies, topologies, and branch lengths were optimised, and the best algorithm for nearest neighbour interchange (NNI) and subtree pruning and regrafting (SPR), was selected. Branch robustness was analysed by approximate likelihood-ratio test (aLRT) [97]. Finally, BioEdit software was used to estimate the identity matrix among alignment sequences (http://www.mbio.ncsu.edu/bioedit/bioedit.html).

### Proteins modeling

The *C. draco’s* 3D protein structures and their orthologs used as references for the case of the (S)-corytuberine synthase (CTS) enzyme (showed in Fig. 5), were modelled by the rigid body grouping method, using the SWISS-MODEL workspace ([98]; http://swissmodel.expasy.org/). The structure of CY17A1 protein (PDB entry 6b82.1), available in Protein Database [99], was used as a template. Each model generated was checked for various parameters that include Z, GMQE (Global Model Quality Estimation) and QMEAN (Qualitative Model Energy ANalysis) scores to assess the accuracy of the model. Once proteins were independently modeled, all of them were superimposed using SWISS-PDB viewer v4.1.0 program [100].

### Putative identification of Taspine and Magnoflorine precursors by liquid chromatography-high resolution mass spectrometry (LC-HRMS)

Leaf, stem, root, and flower samples (*n* = 3 per tissue) were lyophilized in a freeze dryer (Labconco FreeZone®). The sample corresponding to inflorescence (inflorescence with floral stem) was not included in the phytochemical analysis as removal of the flowers from the floral stem results in a leafless flowering stem that we considered likely to be similar to stems or flowers, both of which were analysed separately. Fruits were not included because, like flowers, they are seasonal organs, the abundance of which depends on pollination frequencies. Plant extracts were prepared using an accelerated solvent extraction (ASE) system (ASE 350, Dionex, Thermo Scientific). Briefly, 3 g of dry material were mixed with 1 g of diatomaceous earth and placed in a 34 mL Dionex stainless-steel cell (2.9 cm diameter). The cell was filled with methanol to a pressure of 10.342 MPa and heated to 60 °C for 5 min. Then the cells were washed with methanol (HPLC grade, Sigma-Aldrich) up to 30% of the cell volume and, to avoid bias, a single sample from each organ (1 mL) was prepared by pooling the methanolic extracts of the three replicate samples. A Waters Class I UPLC coupled to a Synapt G2-Si HDMI mass spectrometer was used as analytical platform. 2 μL of each sample extract pool obtained by ASE system was injected in a Waters Acquity BEH (1.7 μm, 2.1 × 50 mm) column. The sample and column temperatures were 15 and 40 °C, respectively. Water (A) and acetonitrile (B), both with 0.1% formic acid (MS grade, Sigma-Aldrich), were used as mobile phase. The solvent gradient was 0–13 min, 1–80% B; 13–14 min, 80% of B; 14–15 min 80–1% of B with a constant flow rate of 0.3 mL/min. It was used an electrospray ionization source in positive mode with a capillary, sampling cone and source offset voltages of 3000, 40 and 80 V, respectively. The source and desolvation temperatures were 100 and 20 °C, respectively. The desolvation gas flow was 600 L/h, and the nebulizer pressure was 0.65 MPa. As lock mass a mixture of leucine-enkephalin (556.2771, [M + H]^+^) it was used and settings were: Mass range 50–1200 Da, function 1 CE, 6 V, function 2 CER 10–30 V, with a scan time of 0.5 s. MassLynx software (v4.1, Waters™) was used to analyse the generated spectrometric information. The tentative identification of taspine and magnoflorine and their precursors such as (S)-reticuline, (S)-corytuberine and magnoflorine methine was performed based on the analysis of accurate mass spectra and fragmentation patterns (maximum error allowed was 3 ppm) with those reported in METLIN-Scripps databases (https://metlin.scripps.edu/).

### Quantification of Taspine and Magnoflorine by liquid chromatography-mass spectrometry (LC-MS)

Quantification and identity confirmation of magnoflorine and taspine were performed in a tandem LC-MS platform. The same samples used for the LC-HRMS assays were analysed but an additional methanolic extract was generated from *C. draco* latex which was harvested from small incisions at intervals of 5–10 cm on branches and on the bark of the main trunk. The latex was lyophilized, and a 1 mg was dissolved in 1 mL of methanol with 0.1% of formic acid (MS grade, Sigma-Aldrich) to analyse by LC-MS-MS. Chromatographic separation and detection of taspine and magnoflorine was performed using an Agilent Technologies 1290 UPLC coupled to a 6460 triple quadrupole (QqQ) mass spectrometer with a Jetstream electrospray (ESI) source. The column used was an Agilent Zorbax SB-C18, 2.1 × 50 mm, 1.8 μm and the column temperature was 40 °C. The mobile phases were water (A) and acetonitrile (B), both with 0.1% formic acid (MS grade, Sigma Aldrich) at a flow rate of 0.3 mL/min. The solvent gradient was 0–10 min, 5–30% of B; 10–11 min, 30–95% of B; 11–12 min, 95% of B and finally 12–14 min, 95–5% of B and remained unchanged for 1 min. The injection volume was 1 μL and each extract pool was injected in triplicate. The mass spectrometer settings were gas temperature 300 °C with flow of 5 L/min. The nebulizer pressure was set at 0.31 MPa, and the sheath gas temperature and flow were 250 °C and 11 L/min, respectively. The capillary and nozzle voltages used were 3500 and 500 V, respectively. The polarity used for the analysis was positive with a fragmentor voltage of 135 V, and a collision energy of 10 V. The acquisition method used was dynamic multiple reaction monitoring (dMRM) with the transitions 342.1 > 296.7 and 370.1 > 325.1 for magnoflorine and taspine, respectively. The magnoflorine commercial standard was purchased from Sigma-Aldrich and taspine was purified in-house by using the protocol described previously [17]. A calibration curve was prepared for each compound with 10 calibration points (0.25, 0.5, 1, 3, 5, 7, 9, 11, 13, 15 and 17 μM). Each calibration point was injected twice, and the areas were plotted against concentration. Quadratic regression was performed to obtain an r^2^ value of > 0.99 for each compound and quantities were established by using MassHunter Workstation Software vB.06.00 (Agilent Technologies). The results are expressed as μg/g of sample (dry weight).

### Laticifer cells abundance in the bark from roots and stems

In order to analyse if the taspine content in distinct plant organs is directly related to the abundance of laticifer cells, we quantified the density of laticifer cells in the roots and the bark on stems. We analysed these organs because their abundance in the distinct organs differs depending on the plant species [2, 101, 102]. In species of the genus *Croton*, numerous laticifer cells are distributed throughout the cortical region of the roots and the bark of stems [1], from which latex is harvested. Samples collected from the same trees mentioned above (see plant material section), were treated with plant fixative FAA (formalin: glacial acetic acid: 70% ethanol, 5:5:90, by volume), dehydrated through a series of ethyl-alcohol solutions and embedded in paraffin (melting point 54–56 °C) [103]. Transverse and tangential longitudinal sections of 12–15 μm thickness were obtained with a rotary microtome and stained with safranin and fast-green [103]. For permanent preparations, a synthetic resin mounting fluid was used. A Nikon Eclipse E600 microscope equipped with a digital camera (Canon Eos Rebel t3i 600D) was used to acquire stacked images. Around 15 distinct images were used to determine laticifer densities.

## Supplementary information

**Additional file 1: Table S1.** Summary of sequencing data generated from *Croton draco*.

**Additional file 2:.** Unigene sequences longer than 1000 bp. (FASTA 51035 kb)

**Additional file 3:.** Unigene sequences shorter than 1000 bp. (FASTA 67268 kb)

**Additional file 4: Figure S1.** Frequencies of *C. draco* unigenes length distribution. **Figure S2.** *C. draco* transcriptome mapped onto KEGG global metabolic network, **Figure S3.** Gene Ontology (GO) terms enriched with a significant number of preferentially expressed genes and identified in each of analysed organs (leaves, stems, roots, fruits, and inflorescences with and without floral scape), **Figure S4.** RT-qPCR validation of RNA-Seq data, **Figure S5.** Biosynthetic pathway of magnoflorine and morphine, **Figure S6.** Putative identification by ion search and HRMS analysis of magnoflorine, taspine and some of their intermediates (PPTX 4505 kb)

**Additional file 5: Table S2.** Annotation of *C. draco* non-redundant (nr) unigenes.

**Additional file 6: Table S3.** Functional categorization of *C. draco* unigenes.

**Additional file 7: Table S4.** Expression profile matrix of *C. draco* unigenes. **Table S5**. Expression profiles of preferentially expressed genes. **Table S6**. OrthoMCL-defined protein families (orthogroups).

**Additional file 8: **Matrices of percentage identity of every pair of proteins identified as orthologs to the reference proteins: tyrosine decarboxylase (**Table S7**, (S)-norcoclaurine synthase **Table S8** and **Table S9**), (S)-norcoclaurine 6′-O-methyltransferase **Table S10**), (S)-coclaurine N-methyltransferase (**Table S11**), (S)-N-methylcoclaurine 3′-hydroxylase (**Table S12**), (S)-corytuberine synthase (**Table S13**).

**Additional file 9: Table S14**: Putative identification of magnoflorine and taspine, and some of their immediate precursors identified by ion search and HRMS analysis. **Table S15**. Primers used in RT-qPCR assays

## Data Availability

All data generated or analysed during this study are included in this published article and its supplementary information files.

## Abbreviations

4OMT: (S)3′-hydroxy N-methylcoclaurine 4′-O-methyltransferase
6OMT: (S)-norcoclaurine 6′-O-methyltransferase
aLTR: Approximate likelihood-ratio test
CNMT: (S)-coclaurine N-methyltransferase
CTS: (S)-corytuberine synthase
FDR: False discovery rate
FPKM: Fragments per kilobase per million
GO: Gene ontology
GTR: General time reversible
HC: Hierarchical clustering
LC-HRMS: Liquid chromatography-high resolution mass spectrometry
LC-MS-MS: Liquid chromatography-tandem mass spectrometry
ML: Maximum likelihood
ncRNAs: Non-coding RNAs
NCS: (S)-norcoclaurine synthase
NMCH: (S)-N-methylcoclaurine 3′-hydroxylase
NNI: Nearest neighbour interchange
PCA: Principal component analysis
RNMT: reticuline N-methyltransferase
RT-qPCR: Reverse transcriptase quantitative PCR
SPR: Subtree pruning and regrafting
TPM: Transcripts per million
TYDC: tyrosine decarboxylase
UTRs: Untranslated regions
