# Supplementary material for: Novel findings to the biosynthetic pathway of magnoflorine and taspine through transcriptomic and metabolomic analysis of Croton draco (Euphorbiaceae)
Source: BMC Plant Biol. 2019 Dec 18;19:560. doi: 10.1186/s12870-019-2195-y (PMC6921603; doi:10.1186/s12870-019-2195-y)
Supplement: Supplementary file 4 — Additional file 4: Figure S1. Frequencies of C. draco unigenes length distribution. Figure S2. C. draco transcriptome mapped onto KEGG global metabolic network, Figure S3. Gene Ontology (GO) terms enriched with a significant number of preferentially expressed genes and identified in each of analysed organs (leaves, stems, roots, fruits, and inflorescences with and without floral scape), Figure S4. RT-qPCR validation of RNA-Seq data, Figure S5. Biosynthetic pathway of magnoflorine and morphine, Figure S6. Putative identification by ion search and HRMS analysis of magnoflorine, taspine and some of their intermediates (PPTX 4505 kb) [file 12870_2019_2195_MOESM4_ESM.pptx]

## Slide 1
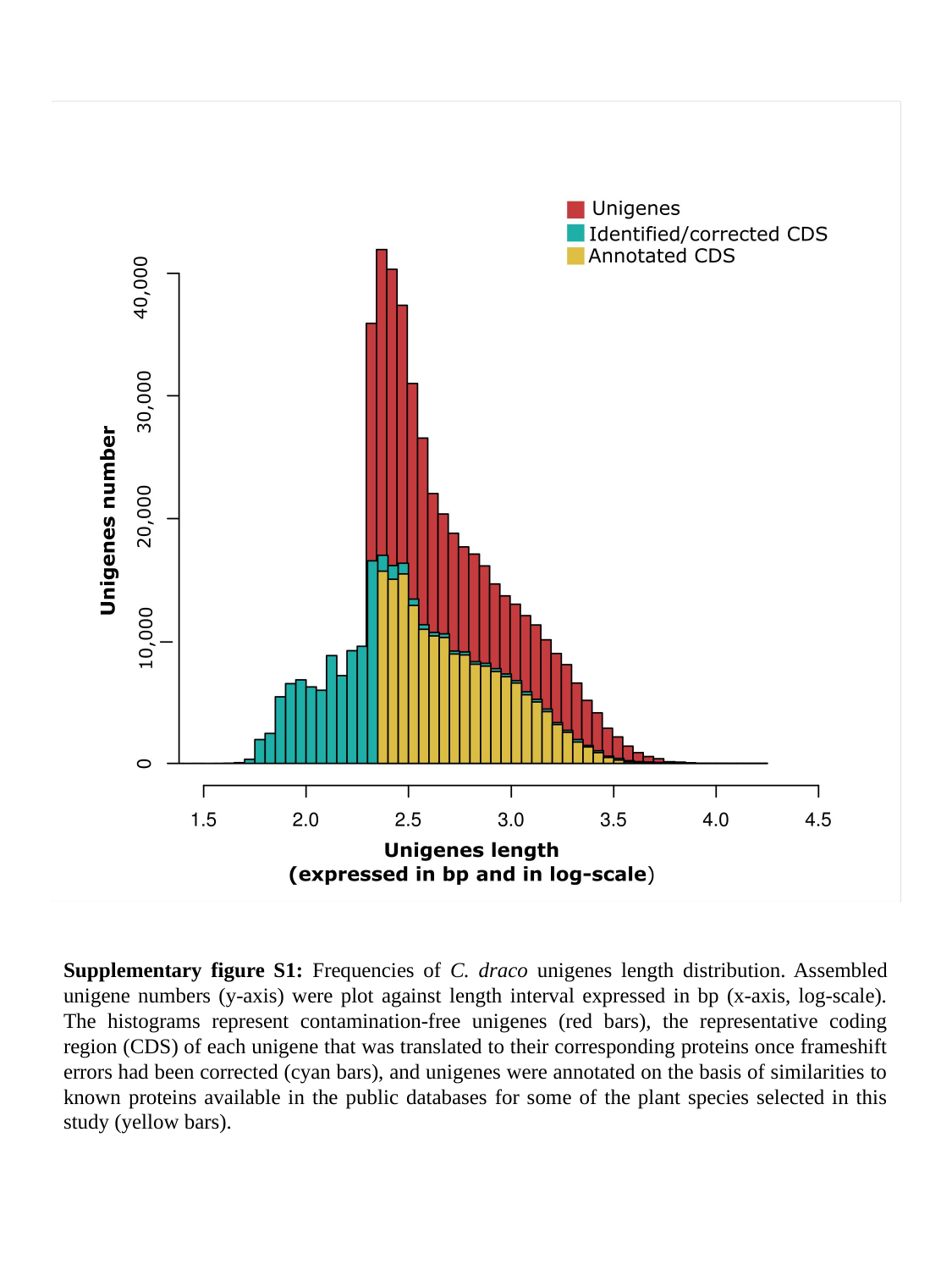

Supplementary figure S1: Frequencies of C. draco unigenes length distribution. Assembled unigene numbers (y-axis) were plot against length interval expressed in bp (x-axis, log-scale). The histograms represent contamination-free unigenes (red bars), the representative coding region (CDS) of each unigene that was translated to their corresponding proteins once frameshift errors had been corrected (cyan bars), and unigenes were annotated on the basis of similarities to known proteins available in the public databases for some of the plant species selected in this study (yellow bars).

## Slide 2
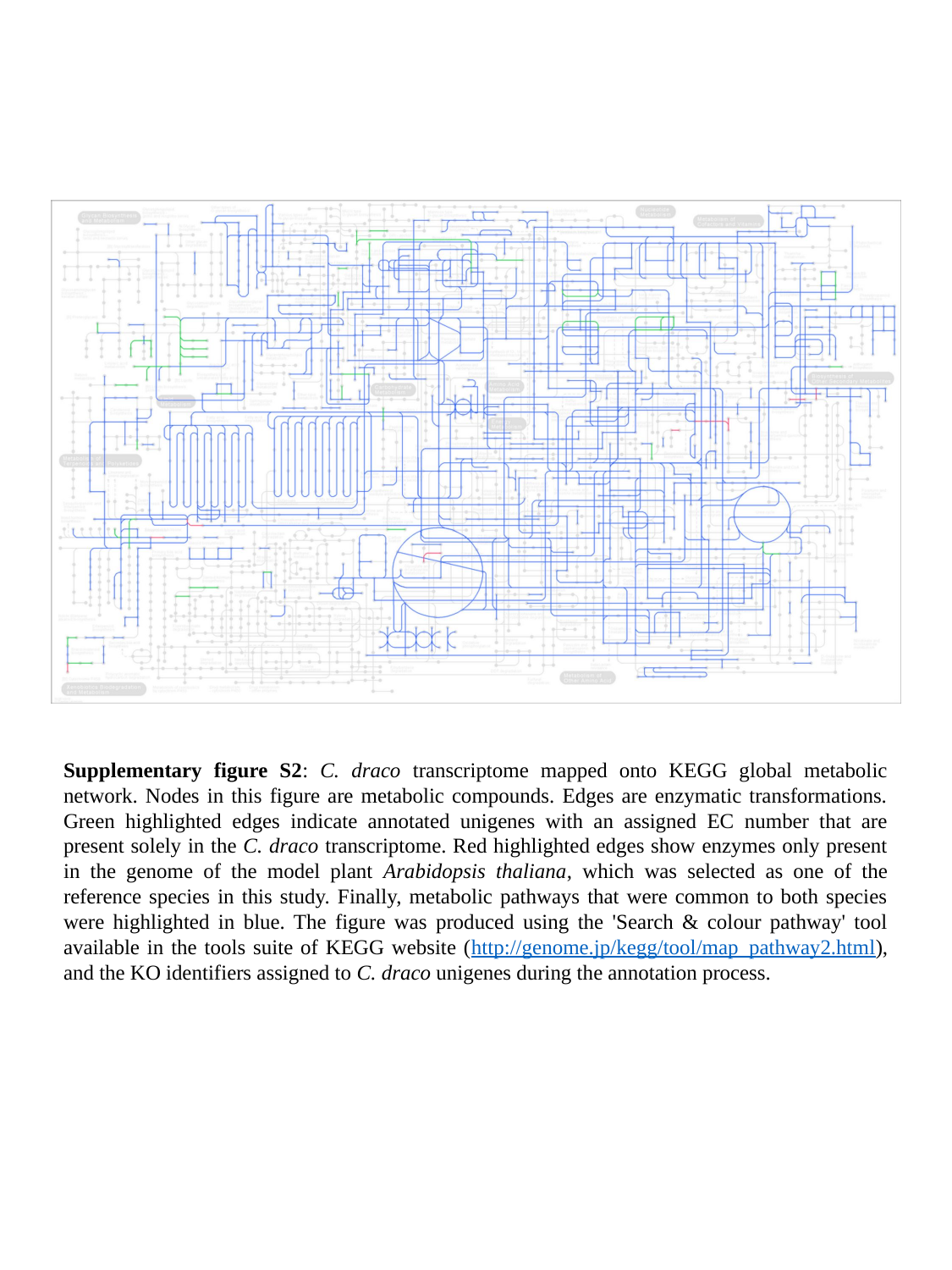

Supplementary figure S2: C. draco transcriptome mapped onto KEGG global metabolic network. Nodes in this figure are metabolic compounds. Edges are enzymatic transformations. Green highlighted edges indicate annotated unigenes with an assigned EC number that are present solely in the C. draco transcriptome. Red highlighted edges show enzymes only present in the genome of the model plant Arabidopsis thaliana, which was selected as one of the reference species in this study. Finally, metabolic pathways that were common to both species were highlighted in blue. The figure was produced using the 'Search & colour pathway' tool available in the tools suite of KEGG website (http://genome.jp/kegg/tool/map_pathway2.html), and the KO identifiers assigned to C. draco unigenes during the annotation process.

## Slide 3
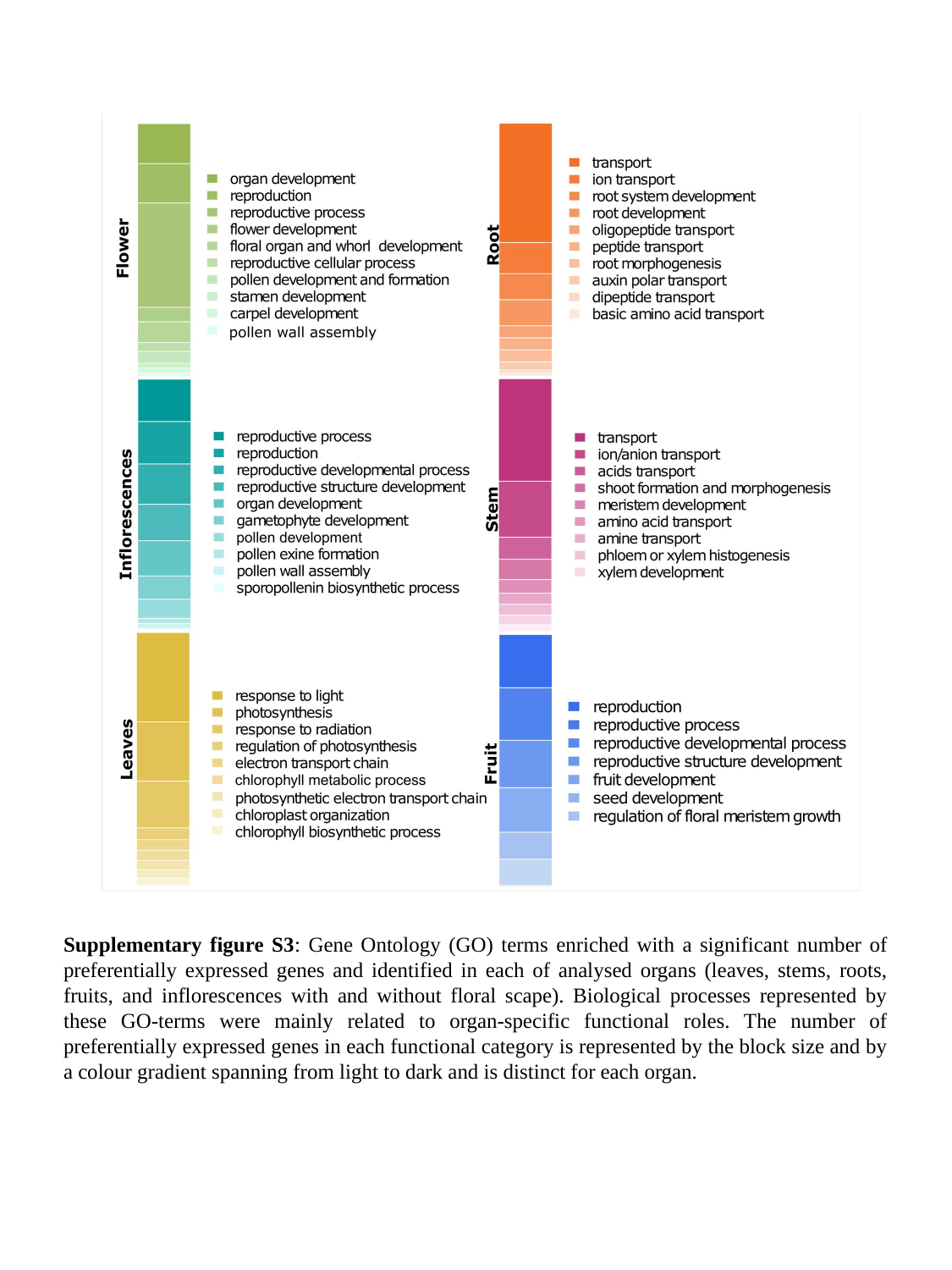

Supplementary figure S3: Gene Ontology (GO) terms enriched with a significant number of preferentially expressed genes and identified in each of analysed organs (leaves, stems, roots, fruits, and inflorescences with and without floral scape). Biological processes represented by these GO-terms were mainly related to organ-specific functional roles. The number of preferentially expressed genes in each functional category is represented by the block size and by a colour gradient spanning from light to dark and is distinct for each organ.

## Slide 4
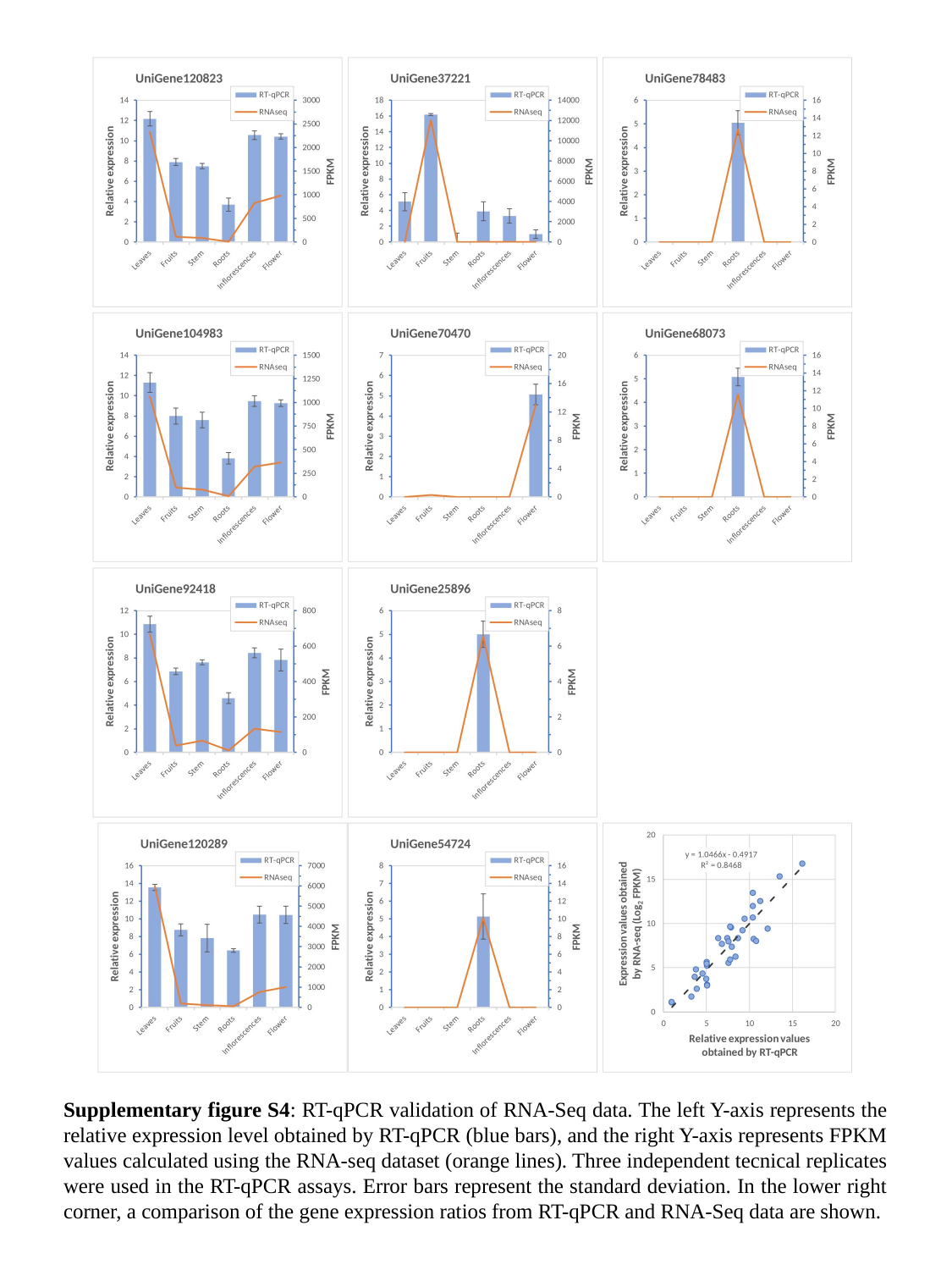

Supplementary figure S4: RT-qPCR validation of RNA-Seq data. The left Y-axis represents the relative expression level obtained by RT-qPCR (blue bars), and the right Y-axis represents FPKM values calculated using the RNA-seq dataset (orange lines). Three independent tecnical replicates were used in the RT-qPCR assays. Error bars represent the standard deviation. In the lower right corner, a comparison of the gene expression ratios from RT-qPCR and RNA-Seq data are shown.

## Slide 5
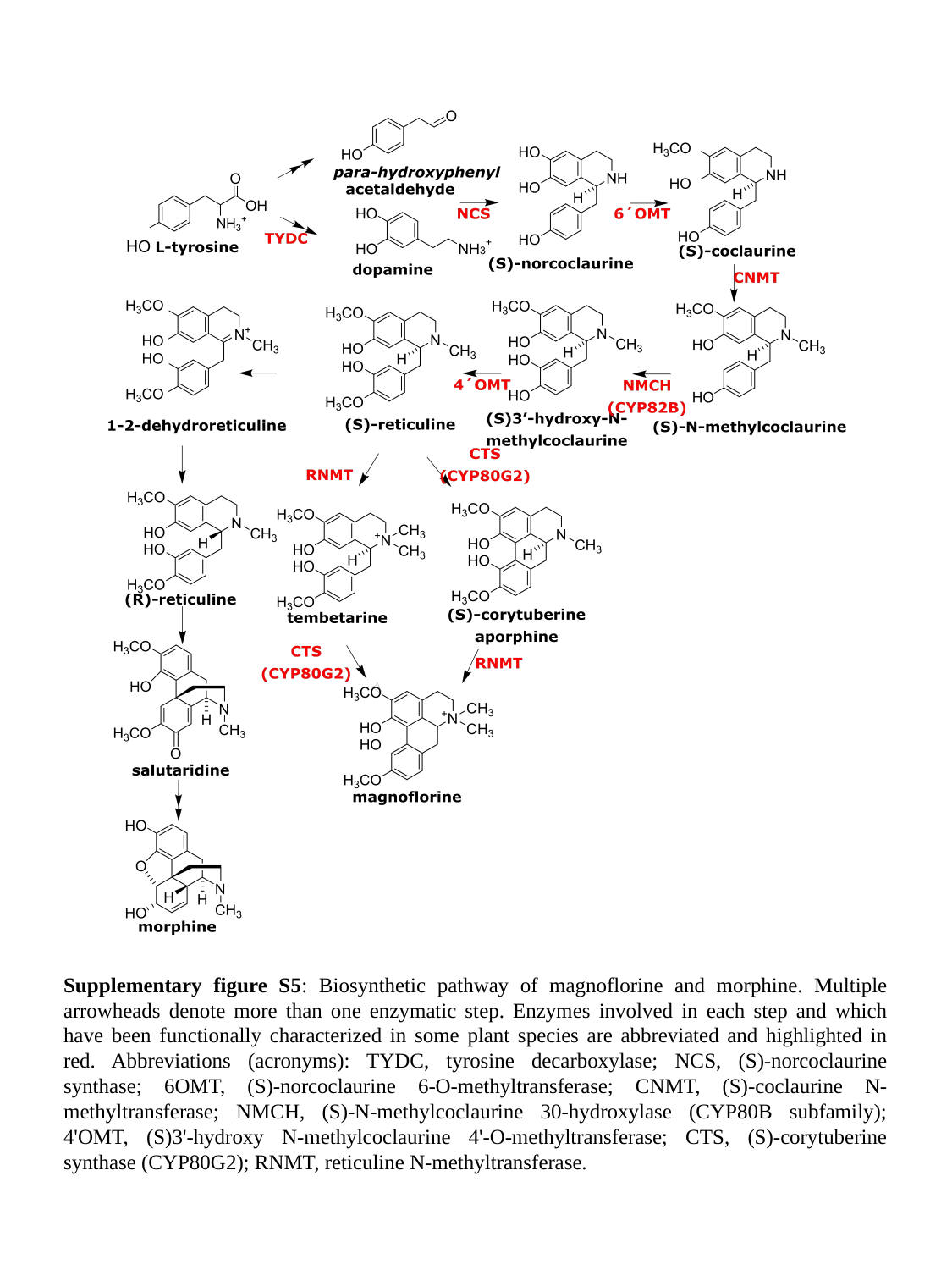

Supplementary figure S5: Biosynthetic pathway of magnoflorine and morphine. Multiple arrowheads denote more than one enzymatic step. Enzymes involved in each step and which have been functionally characterized in some plant species are abbreviated and highlighted in red. Abbreviations (acronyms): TYDC, tyrosine decarboxylase; NCS, (S)-norcoclaurine synthase; 6OMT, (S)-norcoclaurine 6-O-methyltransferase; CNMT, (S)-coclaurine N-methyltransferase; NMCH, (S)-N-methylcoclaurine 30-hydroxylase (CYP80B subfamily); 4'OMT, (S)3'-hydroxy N-methylcoclaurine 4'-O-methyltransferase; CTS, (S)-corytuberine synthase (CYP80G2); RNMT, reticuline N-methyltransferase.

## Slide 6
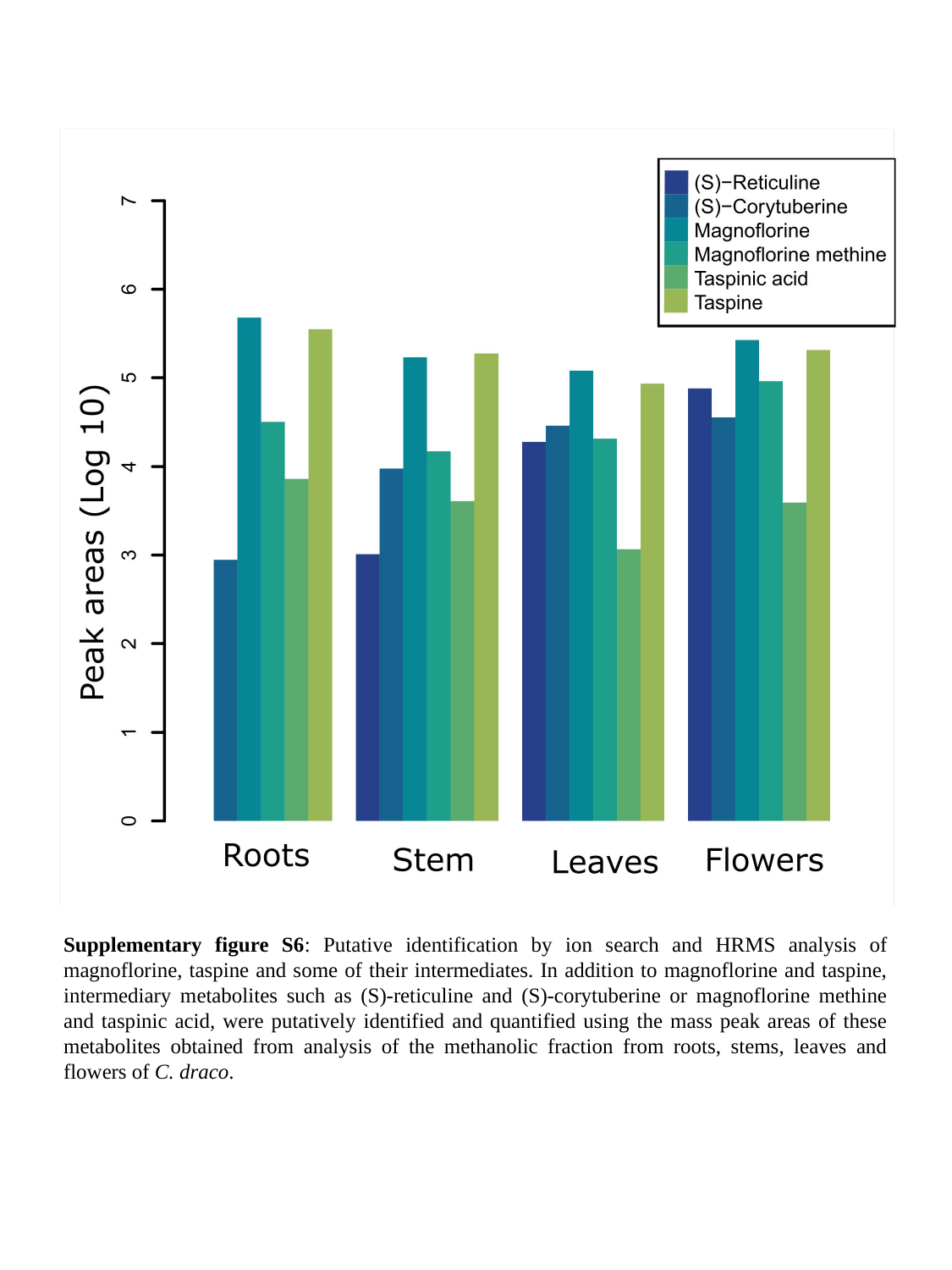

Supplementary figure S6: Putative identification by ion search and HRMS analysis of magnoflorine, taspine and some of their intermediates. In addition to magnoflorine and taspine, intermediary metabolites such as (S)-reticuline and (S)-corytuberine or magnoflorine methine and taspinic acid, were putatively identified and quantified using the mass peak areas of these metabolites obtained from analysis of the methanolic fraction from roots, stems, leaves and flowers of C. draco.
